# Advances in photoacoustic imaging aided by nano contrast agents: special focus on role of lymphatic system imaging for cancer theranostics

**DOI:** 10.1186/s12951-023-02192-8

**Published:** 2023-11-20

**Authors:** Badrinathan Sridharan, Hae Gyun Lim

**Affiliations:** Department of Biomedical Engineering, Pukyong National University, Busan, 48513 Republic of Korea

**Keywords:** Photoacoustic imaging, Lymphatic system, Nanocontrast agents, Cancer theranostics

## Abstract

Photoacoustic imaging (PAI) is a successful clinical imaging platform for management of cancer and other health conditions that has seen significant progress in the past decade. However, clinical translation of PAI based methods are still under scrutiny as the imaging quality and clinical information derived from PA images are not on par with other imaging methods. Hence, to improve PAI, exogenous contrast agents, in the form of nanomaterials, are being used to achieve better image with less side effects, lower accumulation, and improved target specificity. Nanomedicine has become inevitable in cancer management, as it contributes at every stage from diagnosis to therapy, surgery, and even in the postoperative care and surveillance for recurrence. Nanocontrast agents for PAI have been developed and are being explored for early and improved cancer diagnosis. The systemic stability and target specificity of the nanomaterials to render its theranostic property depends on various influencing factors such as the administration route and physico-chemical responsiveness. The recent focus in PAI is on targeting the lymphatic system and nodes for cancer diagnosis, as they play a vital role in cancer progression and metastasis. This review aims to discuss the clinical advancements of PAI using nanoparticles as exogenous contrast agents for cancer theranostics with emphasis on PAI of lymphatic system for diagnosis, cancer progression, metastasis, PAI guided tumor resection, and finally PAI guided drug delivery.

**Graphical Abstract:**

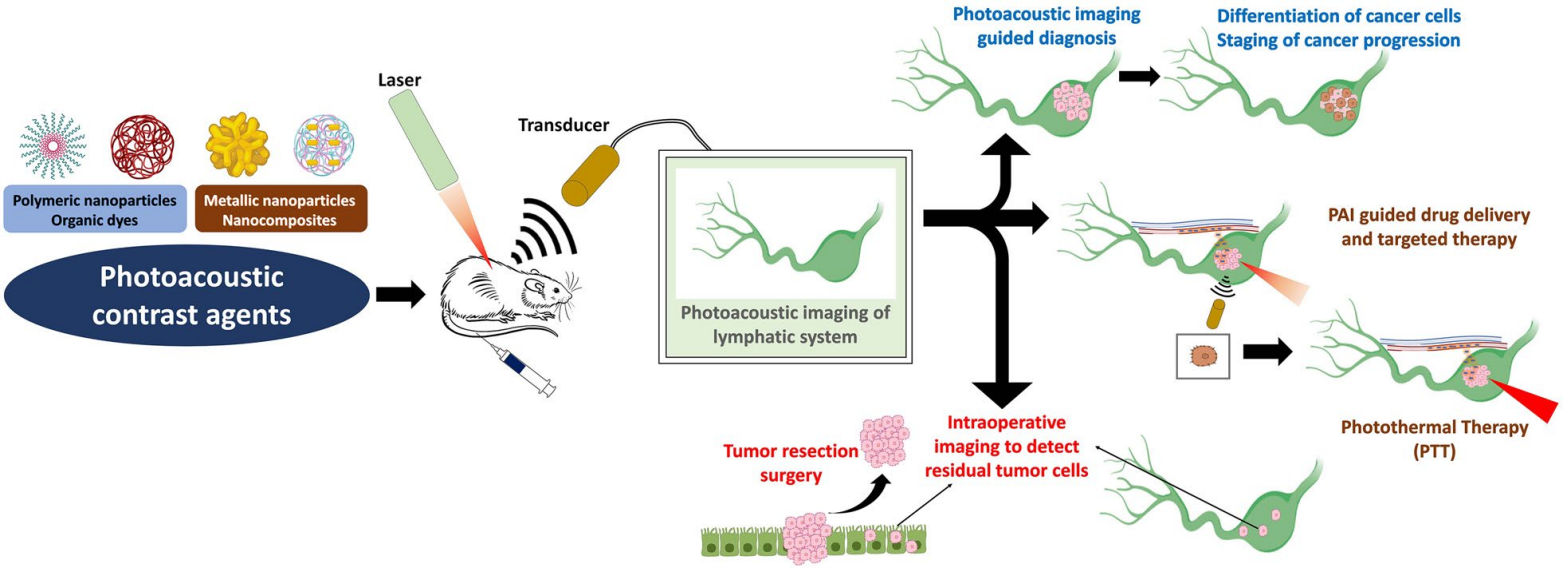

## Introduction

Nanomedicine is an integral part of clinical translation process of diagnosis and therapy. It is a rapidly developing area of research which particularly influence the efficacy and pharmacological stability of the actual drug. It provides liberty to the clinicians to develop patient based, individualized treatment and achieve targeted delivery of a drug that is systemically administered [1]. Constant research in the field of nanomedicine has led to significant developments in nanofabrication methodologies and targeted drug delivery with reduced toxicity. Physical and chemical characteristics of nanomaterials were exploited for its biological applications in management of various diseases at cellular and molecular level for influencing the disease progression. Nanoparticles were used for diagnostic applications for genomic and proteomic analyses, through advanced technologies like nanochip, nanoarray, nanosensors and etc. Nano drug delivery systems provide improved pharmaceutical stability and target specificity of a drug molecules apart from easy access to the intracellular compartment. Nanomaterials have become an integral part of the tissue engineering application and various implants [2–4]. Though the efficacy of nanomedicines are significantly higher than the normal drug, the efficiency can be further improved applying an external force. External stimulus like heat, light or sound, influence and improve every step in nanomedicine application from synthesis to biological efficacy [5]. Nanomaterials are reported to be more stable, readily converted to active form or aid in the treatment process in the presence of external physical stimuli created by NIR/UV light, acoustic waves and thermal stimuli. These results shows that there is a reciprocal relationship between nanomaterials and medical imaging methodologies, where nanomaterial enhance quality of medical imaging and in return they aid nanoformulations in achieving better drug specificity and efficacy. The principle of ultrasound (US) and acoustic vibrations were well studied and has been applied in different methods for imaging the treatment processes [6].

**Fig. 1:**
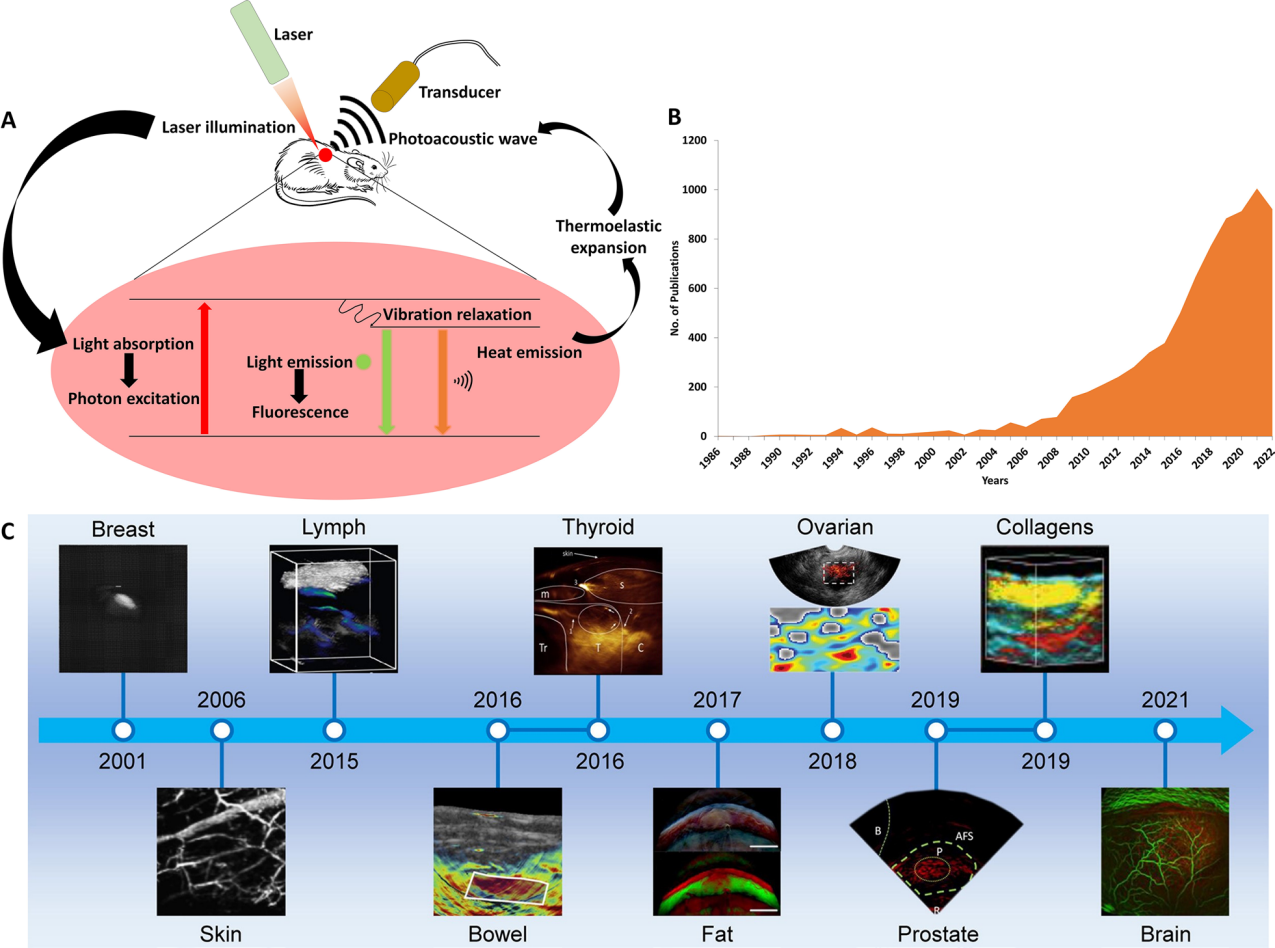
**A** Basic principle of Photoacoustic wave generation; **B** year wise number of publications related to nanoparticles and photoacoustic imaging; **C** advances in PAI for different organs in vivo Part **a** of the figure was inspired from the article published by Jeong, Kim and co-workers in Biosensors journal in the year 2022 [36]. Part **b** of the figure was prepared using R studio with Biblioshiny package for all the analysis for downloaded data. We have imported the acquired Scopus plain text record in Bibliometrix R web interface. Part **c** of the PAI images were reproduced from the different articles published and are cited here [27, 37–46]

Photoacoustic imaging (PAI) is one of the application methods among ultrasound technologies which is a combination of light irradiation and ultrasound detection (Fig. 1a). The basic principle of PAI involves irradiation of laser which was pulsed for a short duration of time and the molecules in the specimen absorbs light energy. The absorbed light excites the molecules, which return back to the ground state be releasing energy in the form of fluorescence and heat. The thermal energy release induces thermoelastic expansion of the surrounding tissues that produces acoustic vibration which is termed as photoacoustic waves and this is received by ultrasound transducer [7, 8]. Evidences suggest that, images acquired with light-in and sound-out principle resulted in imaging tissues to a greater depth, compared to images obtained from light-in and light-in principle. Though the image resolution from the latter is higher, the penetration depth beyond 1 mm can be achieved only by PAI [9]. Understanding of the physics behind photoacoustic imaging goes back to 1880 where Alexander Graham Bell observed photo acoustic (PA) effect when sound was generated after absorption of sun light [10]. The research focus on PA effect was sporadic and only in the late 1970s, its importance was realized and applied in various research fields [11]. Despite numerous advancements in PA based technologies, their clinical translation of in management of complex diseases is still a formidable task for the experts. Neurological disorders, breast cancer, skin lesions and lymphatic imaging are some of well-established imaging applications of PA systems. On the other hand, PAI has been instrumental in image guided biopsy, drug delivery and surgery [12]. Clinical studies with PAI were conducted and successfully reported for diagnosis of breast cancer, skin lesions, musculoskeletal disorders, inflammation mediated disorders like cancer, rheumatoid arthritis and certain vascular complications [13–15]. In this review we have discussed about the emergence of nanomedicine and its influence in improvisation of photoacoustic applications and vice versa. Then we have briefed about the importance of lymphatic system and its advantages as a best route for drug administration compared to others. The studies exhibiting the importance of lymphatic circulation in cancer progression was reviewed. Then we have reported the literature evidences on how the PAI of lymphatic system is an integral part of cancer theranostics including inevitable role of nanomedicine.

## Various imaging modalities based on photoacoustic effect

There are different methodologies that are developed with photoacoustic principle such as photoacoustic tomography (PAT), photoacoustic microscopy (PAM), and multispectral optical tomography (MSOT) [16]. Photoacoustic microscopy involves point by point scanning method of PAI and the data is acquired by spherical transducers which was provided as images without Arificial intelligence (AI) based reconstruction [17]. The advantage of PAM is its robust imaging method that can visualize the tissues without contrast agent and utilized endogenous contrast agents for signal enhancement. The optical or acoustic foci in PAM will be scanned into imaged obtained from depth-resolved signals and is primarily aimed at acquiring data with high-resolution and rather than focusing on deeper penetration. This methodology was further classified as optical resolution PAM, which has more tighter optical focusing while with more tighter acoustic focusing is employed in acoustic resolution PAM. Optical resolution PAM has the lateral resolution of up to 10 μm and penetration depth is very low at 1.5 mm. The acoustic resolution PAM has the lateral resolution of 45 μm and 11 mm of tissue penetration depth [18, 19]. On the other hand, to resolve the issue of deeper organ imaging, photoacoustic endoscopy was developed which consists of miniatured transducer that records the acoustic signals obtained from thermoelastic expansion of the target organs and digitalized for image reconstruction. PAE is a smaller version of PAM, and can be employed as acoustic resolution-PAE and optical resolution PAE. the penetration depth of PAE is increased up to ~ 1.5 mm, which is utilized for imaging vital internal organs, especially the Gastrointestinal (GI) tract and vascular imaging for atherosclerotic plaques [20–22]. PAT on the other hand works with ultra-short laser pulses and combines the US and optical imaging with higher resolution (250 μm) for better imaging process with a penetration depth of up to 70 mm with minimal invasiveness [12, 23–25]. Though lateral resolution is not very appreciable, PAM has several advantages like imaging tumor in deeper locations, high frequency laser was used to reduce the scan time and obtain high quality images and it has an accommodating instrumentation, where other modalities can be combined for better image-based information of the disease state. MSOT is upgraded version of PAT where sample was illuminated with laser from multiple wavelengths and receiving the US signal with or without contrast agents. The obtained US signal was reconstructed computationally by spectral un-mixing to separately visualize the emitters in the target tissues. The macroscopic MSOT can image with penetration depth of greater than 10 mm (100–50 μm lateral resolution), while mesoscopic MSOT show penetrating depth up to 10 mm (1–50 μm lateral resolution). MSOT aid in anatomical imaging, dynamic/functional imaging to analyse the hemodynamics, motion artifacts, drug uptakes, molecular imaging for tissue oxygenation state, fluorescent tagging for protein and other intracellular molecular imaging. Advantages of MSOT involve differentiate oxygenation state of Hemoglobin (Hb), exogenous contrast agents with varies absorption maxima can be used, reduced imaging time and the most interesting feature of MSOT is the ability to image and provide on the pharmacokinetics of drug [16, 26].

## Technological advances in PAI based imaging systems

Technological advances in PAI and pertaining instrumentations are remarkable over the years (Fig. 1C). Established companies like iThera Medical GmbH, FUJIFILM VisualSonics, CalPACT/Union Photoacoustic Technologies, Mindray Bio-Medical Electronics, Guangdong Photoacoustic Medical Technology, etc. has shown great interest in development of photoacoustic systems for clinical imaging based disease management [27]. In the year 2006, PAM was utilized for skin and vascular structure of the subcutaneous region. This has been the driving force for the experts to emphasize their research on improving the PAM and PA mesoscopic imaging (Raster scan optoacoustic mesoscopy) for better diagnosis of skin ailments such as atopic dermatitis and skin sensitivity. Though in the subsequent years PAI instrumentation was under constant development for imaging deeper organs, it should be noted that both PAM and PA mesoscopy were unable to show imaging depth beyond the skin barrier. Nonetheless, PACT showed better imaging depth and speed for imaging percutaneous organs during initial days of development and later was successful in imaging breast tissues, lymphatic systems, intestine, ovaries, prostate and etc. [12, 27]. Multispectral optoacoustic tomography developed by iThera Medical GmbH can unmix the PA spectrum obtained from the endogenous chromophores like hemoglobin, melanin, lipids, etc. Breast cancer imaging with PAI platforms has been significantly successful and instrumental in improved management. FDA has approved the diagnosis of breast cancer with the PACT device developed by Seno Medical, USA [16]. Spectral analysis technology had an influence on unmixing the PA spectra of melanin, lipids, collagen and etc., which is differentiated based on their absorption co-efficient at different wavelength and it is termed as quantitative chromophore analysis. Spectral unmixing has become vital for diagnosis of melanoma, muscular disorders, cardiovascular complications, and skin diseases [28]. Apart from the anatomical information of the organs, the functional status was also diagnosed with PAI. Photoacoustic viscoelasticity provides the mechanical characteristics of the tissues through the phase difference relative to excitation and measurement of the rise time of acoustic signals corresponding to displacement. In case of cancer lesion, the PAI systems rely on the oxygen requirement with in the microenvironment to distinguish tumor tissues from normal tissues especially when the anatomical structures are undifferentiable [29, 30]. The success of PAI in a clinical set up, encounters specific challenges during imaging process like difference in speed and velocity of sound, inconsistent optical fluence and optical attenuation due to difference in wavelength. Considerable amount of research has gone into interleaved spectroscopic compensation for wavelength dependent laser fluence for addressing these issues [31–33]. During clinical PAI, motion artifacts due to involuntary movements such as heart beat, breathing, peristalsis and etc., are some of the major concerns that greatly affects the image resolution and output of quantitative analysis. Collaboration with specialists in AI and informatics has been beneficial in developing algorithms such as synthetic surface-based algorithms, demons-based tracking, multi-scale vascular feature mating method, deep learning-based algorithms, intensity phase tracking, ultrasound-guided motion correction [27, 32, 34, 35].

## Success of PAI in clinical diagnosis and management

Currently the PACT guided diagnosis of structural and functional abnormalities has become well-established, which mainly depends on analyzing the oxygenation state of Hb and its distribution among the tissues [47]. Since 2001, the development in PAI based diagnosis platform has shown enormous success (Table 1) which includes clinical prototype LOIS-64, hemispherical detector array, multispectral PAT and single breath hold PACT [47, 48]. Han et al., in the year 2021 analyzed the PA images by calculating the optical fluence distribution [49]. Photoacoustic dermoscopy (PAD) is a groundbreaking invention for imaging the through entire depth of the skin that enables the differentiation of melanin in the epidermis, capillary loops in epidermal-dermal junction as well as dermal and subcutaneous vasculatures [50–52]. Pilot studies with PAD showed success with diagnosis of psoriasis, diabetic foot ulcers, melanoma, and other skin ailments [51, 53, 54]. PACT showed improvements in dermatologic complications, especially Tsuge et al., has shown better images of anterolateral thigh perforators. Further, the comparison between the PACT and US demonstrated that, subcutaneous microvasculature were imaged and the resulting images were printed on body attachable sheets that can be used as real time intraoperative navigation of the blood vessels [55].

Dynamic PA imaging enhanced by exogenous contrast agent (indocyanine green—ICG) showed better imaging and analysis of liver fibrosis in mice, where the imaging process aided in functional evaluation of the fibrotic progression [56]. Although endoscopy and tissue biopsy were considered the gold standard for diagnosis of GI complications, chance of potential side effects and limited diagnostic value has led to a void in this approach [57]. However, PAI based imaging technologies such as MSOT and PAE have played a crucial role in bridging the gaps and achieving successful GI diagnosis. MSOT data obtained with six different wavelengths that distinguish various oxygenated states of Hb and its concentration has helped in diagnosis of Crohn’s disease with significant insights into the information on degree of inflammation. The endoscopic images were compared to the MSOT image parameters as confirmatory evidences with a scoring system called simplified endoscopic score for Crohn’s disease (SES-CD). The comparative analysis showed that MSOT-based diagnosis proves to be a more sensitive and dependable approach with less invasiveness [58]. On the other hand, PAE was successful in reporting the colorectal cancer in the clinical setup. PAE is a hybrid instrumentation of PAM and endoscopic ultrasonography and it differentiates the normal rectal tissue architecture, characterized by a layered rectal wall and evenly arranged submucosal layer, and cancerous tissue, which exhibits a disturbed rectal wall and submucosal vascular structure. The convolution neural network model of PAM is very helpful in differentiating the signatures of malignant rectal tissues from normal tissues [59, 60]. Similar to the intestinal disease diagnosis, fine needle biopsy sampling guided by ultrasound is the gold standard for thyroid imaging and inability to view the whole tumor microenvironment is its major drawback [61, 62]. The prospects of PAI in thyroid imaging are significant, since thyroid is located at a depth of 2–3 cm and fall within the imaging range of PAI. Handheld MSOT was the first successful PAI based imaging method that utilizes detector adaptable for imaging the whole neck region [63]. MSOT, along with quantitative analysis of the of fat content, total Hb and deoxy-hemoglobin has played a key role in diagnosis of thyroid disease [64]. MSOT was also reported to aid in collagen imaging by unmixing the endogenous chromophore spectra [65]. Conditions like tissue fibrosis and Duchenne muscular dystrophy were successfully diagnosed with MSOT by NIR illumination and quantitative analysis of collagen. MSOT provides substantial information on molecular features during diagnosis of Duchenne muscular dystrophy compared to MRI. MSOT has also demonstrated its ability to investigate the fat metabolism that could shed lights on the progression certain metabolic disorders like diabetes, obesity and etc. [44].

Ovarian cancer management remains an unresolved challenge for the experts due to lack of diagnostic accuracy in its early stages of the progression while imaging tools like MRI, CT and PET are helpful primarily during the surgical process. Transvaginal co-registered PACT and pulse-echo ultrasound imaging were used together to obtain the total Hb concentration and mean oxygen saturation of the ovarian tissues (with/without lesions) [37, 66, 67]. Metastatic tumors demonstrated high vascularity and showed low oxygen saturation compared to the benign or normal ovaries. The study showed the ability of PAI technology to diagnose tumors at an early stage of progression using vascular parameters. Prostate cancer is one of the common non cutaneous cancer predominant in men which was diagnosed previously by transrectal ultrasound (TRUS) by visualizing the prostate anatomy and aided in needle biopsy process. However, the diagnostic specificity and information about the physiological status of the prostate tissues and tumor microenvironment was not provided by TRUS. Recently, an integrated set up of TRUS with PA device was developed which helped in observing both the anatomical and functional status of the prostate. The patient diagnosed with prostate cancer via PET-MRI imaging was subsequently examined using TRUS-PA and multispectral contrast provided by Hb and ICG was utilized to achieve the imaging depth of up to 3–4 cm. It was obvious from the output images that combination PA and US has significantly improved the image quality and diagnostic value [27, 38].

**Table 1:** Summary of in vivo photoacoustic human imaging introduced in this review [27]

Human imaging in vivo	Application field	Current clinical techniques	Current clinical deficiencies	Biomarkers of PAI	Advantages of PAI
Breast	Breast tumor	X-ray mammography Ultrasonography MRI	Ionizing radiation Low contrast Expensive	Hb HbO_2_	High resolution Oxygen saturation Elastography
Skin	Skin disease Skin grafting Cosmetics	Dermoscopy RCM Ultrasound	Low penetration Low contrast	Hb HbO_2_ Melanin	High resolution Full-thickness skin Oxygen saturation
Lymph	Sentinel lymph nodes Lymph dissection Lymphangiography	Biopsy Lymphography Lymphoscintigraphy	Repeat biopsy Radiation Complication	ICG	High resolution Vascular/lymphatic-colocalization
Bowel	Crohn’s disease Rectal cancer	Endoscopy Ultrasonography CTE MRE	Low penetration Radiation Low contrast	Hb HbO_2_	Non-invasive Oxygen saturation Endorectal PAUS High diagnostic rate
Thyroid	Cancer Thyroid nodules	Fine needle aspiration Ultrasonography	Low contrast	Hb HbO_2_	High contrast Oxygen saturation
Fat	Fatty tumors	F-FDG-PET/CT MRI	Radiation Expensive	Lipid	Non-invasive Rapid imaging
Ovarian	Cancer	Transvaginal ultrasound	Low contrast	Hb HbO_2_	High contrast Oxygen saturation
Prostate	Cancer	Transrectal ultrasound	Low contrast	Hb HbO_2_ ICG	High contrast Simultaneous TRPA/US Specific imaging
Collagen	Bone health DMD	MRI	Expensive	Collagen	High contrast Non-invasive Rapid imaging
Brain	Brain function	fNIRS fMRI	Low-spatial-resolution Expensive	Hb HbO_2_	High resolution Functional imaging Rapid imaging

*DMD* Duchenne muscular dystrophy, *RCM* reflectance confocal microscopy, *CTE* computed tomography enterography, *F-FDG-PET/CT* fluorodeoxyglucose positron emission tomography/computed tomography, *fNIRS* functional near-infrared spectroscopy, *fMRI* functional magnetic resonance imaging, *TRPA/US* transrectal photoacoustic and ultrasound

**Fig. 2:**
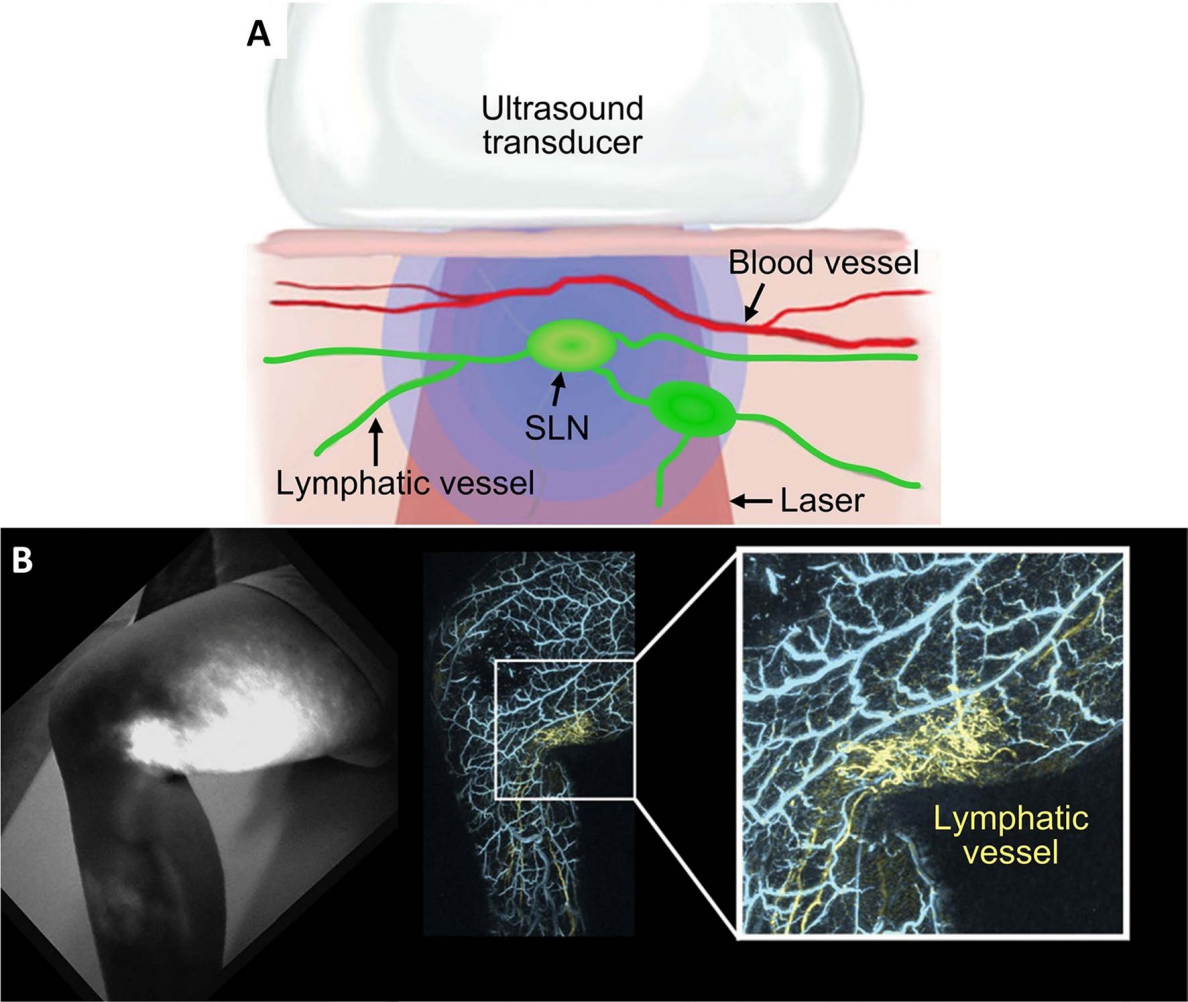
In vivo photoacoustic lymphangiography. **A** Schematic diagram of photoacoustic lymphangiography based on ICG. **B** Photoacoustic lymphangiography evaluates the postoperative patency of lymphaticovenular anastomosis [27, 42, 73]. Copyright © 2023 Science Foundation of China Publication Department, National Natural Science Foundation of China. Publishing Services by Elsevier B.V. on behalf of KeAi Communications Co. Ltd. Copyright © 2021 PRS Global Open

Magnetic resonance imaging (MRI) paved the way for in vivo imaging of functional brain and is commonly referred to as functional MRI (fMRI). fMRI utilizes the blood oxygen level-dependent (BOLD) contrast to map and study brain activity. Challenges like cost of imaging, difficulties in portability and unsuitability for claustrophobic patients are serious [68]. On the other hand PAI utilizes the neurovascular coupling and provide a complete information of the neural activity with the help of endogenous contrast from Hb. Cerebral vascular mapping can be done with PAI and further with MSOT, the blood oxygen saturation and cerebral blood volume can be measured, which was not convincingly achieved by fMRI [68, 69]. Zhang et al., comprehensively studied the angiogenesis, blood brain barrier (BBB) characteristics and microenvironment of glioblastoma in mice and reported that photoacoustic molecular imaging aided in disease staging with high contrast and better visualization of various components compared to observations from other clinical imaging modalities [70]. Recently 1K3D-fPACT was developed for 3D functional brain imaging and it is advantageous over the conventional PACT in terms of field of view and imaging speed. Magnetic resonance angiography (MRA) was compared with PACT and it was observed that MRA is sensitive to arteries, while PACT depends on hemoglobin for the contrast and focuses both arteries and veins. fPACT was reported to play key role in diagnosis of cerebrovascular diseases and has an edge over the other diagnostic tools because of its easy handling techniques, better output with minimal instrumentation [39]. During diagnosis of lymphatic system related disease conditions, observing the lymph nodes and lymphatic circulation is one of the challenging steps. PAI of lymphatic system has gained serious interest in the recent years, especially during diagnosis of breast cancer, imaging the sentinel lymph node (SLN) plays a crucial role in differentiating the tumor cells and metastatic progression [71]. The only drawback in PAI of lymphatic vessels is the lack of endogenous contrast agent like Hb in case of blood vessels. MSOT was more helpful in distinguishing the metastatic and normal lymph nodes with excellent resolution and imaging depth of up to 5 cm [72]. Lymphangiography performed with PAI was significantly helpful in postoperative lymphaticovenular anastomosis for diagnosis of lymphedema (Fig. 2). Utilization of photoacoustic lymphangiography following the ICG administration assists in diagnosis of tumor and pertaining lymphatic complications with more specificity and better image quality [16, 73].

PA technology has gained lot of interest and significant development has occurred since its venture into the clinical arena [74]. Similar to other methodologies, minor drawbacks in the imaging process and the quality of output images have been encountered in PAI. As a result, continuous exploration and research efforts are being carried out to address and improve these limitations. The image acquisition during clinical PAI was significantly aided by endogenous contrast enhancing molecules like Hb, melanin and etc., which specifically has NIR absorbing property. However absence of endogenous contrast agents within the lymphatic system or other deeply seated organs make PAI a challenging task [9]. To address this issue, contrast enhancing molecules like organic dyes, polymers, or metal-based nanoparticles with NIR absorption were administered prior PAI imaging. In the following section the role of nanoparticles in PAI and how nanoparticles influence the upgradation of imaging process and quality of the images obtained [75]. In the last 15 years (since 2007), up to 713 articles (both research and review articles) were published on clinical applications of PAI (Fig. 1B). Research and development with respect to PAI and nanomedicine began to show a rising trajectory since 2007 and it attain significant prominence since last decade particularly from 2013. Among various applications of PAI, diagnosis of cancer through cellular and molecular imaging helps in understanding the tumor microenvironment like angiogenesis [76], oxygenation mapping [24], brain imaging [77], hypoxia [78], cortical blood volume [79] and lymph node metastases [16, 80].

## Nanoparticles and photoacoustic applications

Nanoparticles are highly dynamic materials as their physical and chemical characteristics can be manipulated for improvement of the biological stability and efficacy, irrespective of their chemical composition [81]. Therapeutic properties of nanomaterials were significantly enhanced by the external stimuli and the pertaining research works are ongoing for several decades. As a part of disease management process by nanotherapeutic agents, support from biomedical devices (US, NIR/UV, Laser, MRI, etc.) are inevitable [5]. Concurrently, researchers were also focusing on enhancing the quality of medical imaging and improving diagnostic accuracy by harnessing the nanotechnological advances [82]. Nano-contrast agents represent an emerging group of materials that hold a pivotal role not only in enhancing the quality of output images but also in differentiating overlapping disease symptoms and simplifying the disease staging. Imaging methods like MRI, CT, X-ray, US has become more efficient in disease diagnosis with the help of contrast agent in the nanoform [75, 83]. In case of PA imaging several nanoparticle formulations with varied chemical compositions were reported. The NIR absorption property of the nanomaterial was exploited in this regard and this is the common approach of variety of nanomaterials such as carbon-based nanocomposites, semiconductive polymeric materials in exerting photoacoustic property. Several organic dyes were also used as PAI contrast agent in the native or nanoform [75, 84]. This section discusses about nanoparticles with different chemical compositions and their involvement in PAI mediated disease management.

### Inorganic/metal nanoparticles

**Fig. 3:**
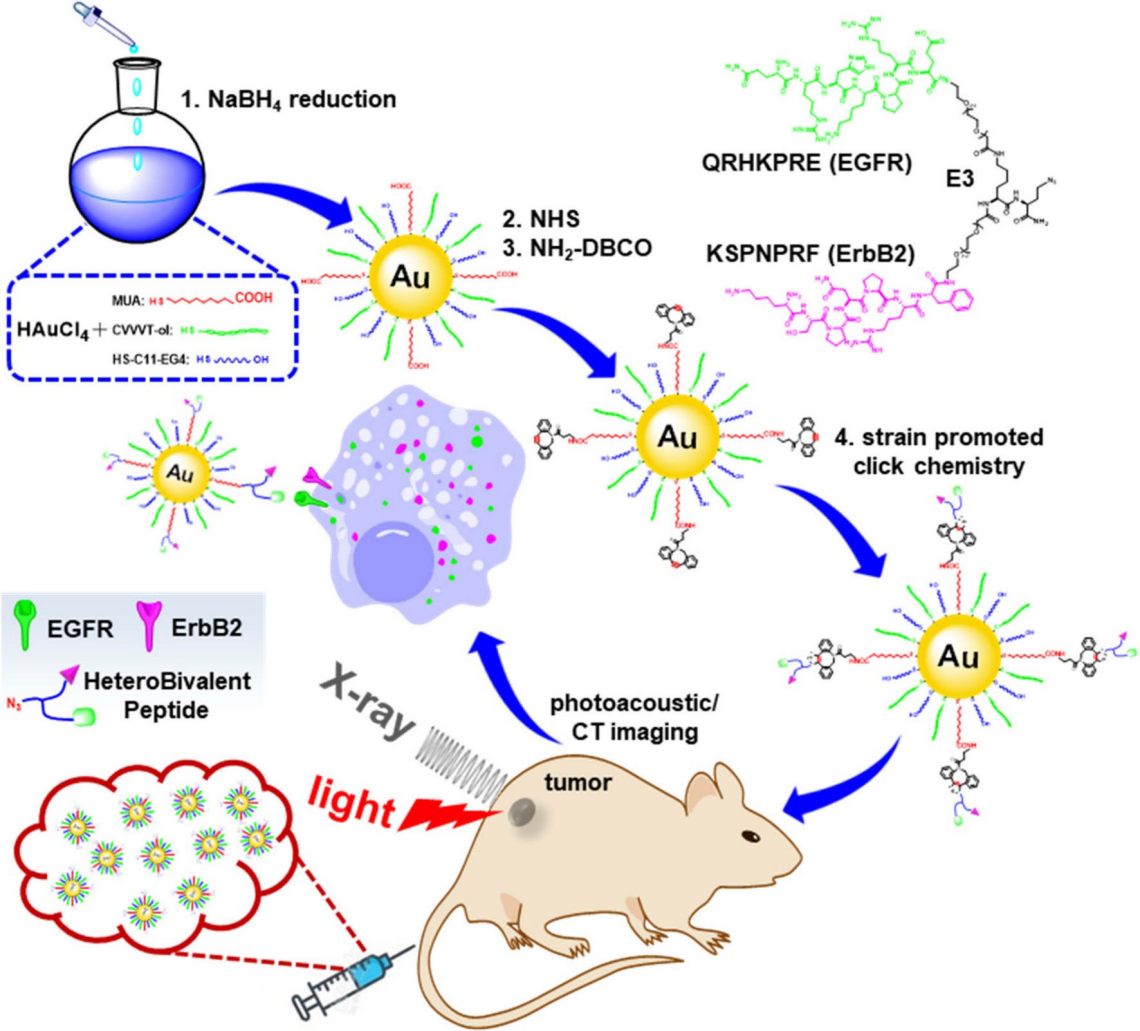
Schematic is shown for labeling the thin layer-protected gold nanoparticles with the heterobivalent (HB) peptide-specific for epidermal growth factor receptors (EGFR) and ErbB2 (HB-Au-NPs) developed for dual photoacoustic/CT imaging. The image was reproduced from article published by Chen, Wang and co-workers in Pharmaceuticals journal in the year 2021 [86]

Metal nanoparticles are the first reported materials with the ability to provide strong PA signals. Among the reported metals, gold nanoparticles showed excellent PA property [7, 85]. Their uniqueness in shape, size and tunable properties makes them suitable candidates for multiple PAI application. Size of the metal nanoparticle plays a significant role in their PA signaling, where the smaller sized particles (< 10 nm) provide low signals compared to the larger particles (20–150 nm) (Fig. 3) [86, 87]. Non-linear PA effects were generated by gold nanoparticles when they were endocytosed after aggregation. After endocytosis of the plasmonic nanoparticles they lead to raise in temperature due to coupling and improved acoustic signals [88]. Similar studies on gold nanoparticles coupled with photosensitive proteins showed that the nanoparticles move apart upon photoactivation of protein resulting in generation of PA signals [89]. Due to poor biodegradation of gold nanoparticles, they were clustered with biodegradable stabilizing agent to obtain a robust PA signals and rapid clearance through kidney [90]. Phospholipid nanoparticles added with gold nanoparticles provided 10 times improved PA signals [91]. Sodium yttrium fluoride doped with ions like ytterbium (Yb^3+^), erbium (Er^3+^) and terbium (Tb^3+^) were reported to convert NIR into visible light, which helps in generation of PA signaling that are tested in small animals [92]. Copper based materials (especially copper sulfide) amplifies the PA signals six times more than the signal obtained from blood. Copper sulfide materials were successful in imaging the breast tissues up to 40 mm deep and also in visualizing the structure of neural networks [93]. Similarly gold nanoparticles conjugated with magnetic iron oxide nanoparticles facilitated multiplex PA imaging and subsequent targeting of cancerous lesions and circulating tumor cells (CTCs). Integrating the magnetic nanoparticles with urokinase plasminogen activator has resulted in specific detection of CTCs with minimal interference from blood cells. Likewise, superparamagnetic iron oxide nanoparticles (SPIONPs) were helpful in photoacoustic tomography when they were coated with silica [94, 95].

### Organic/polymeric nanoparticles

**Fig. 4:**
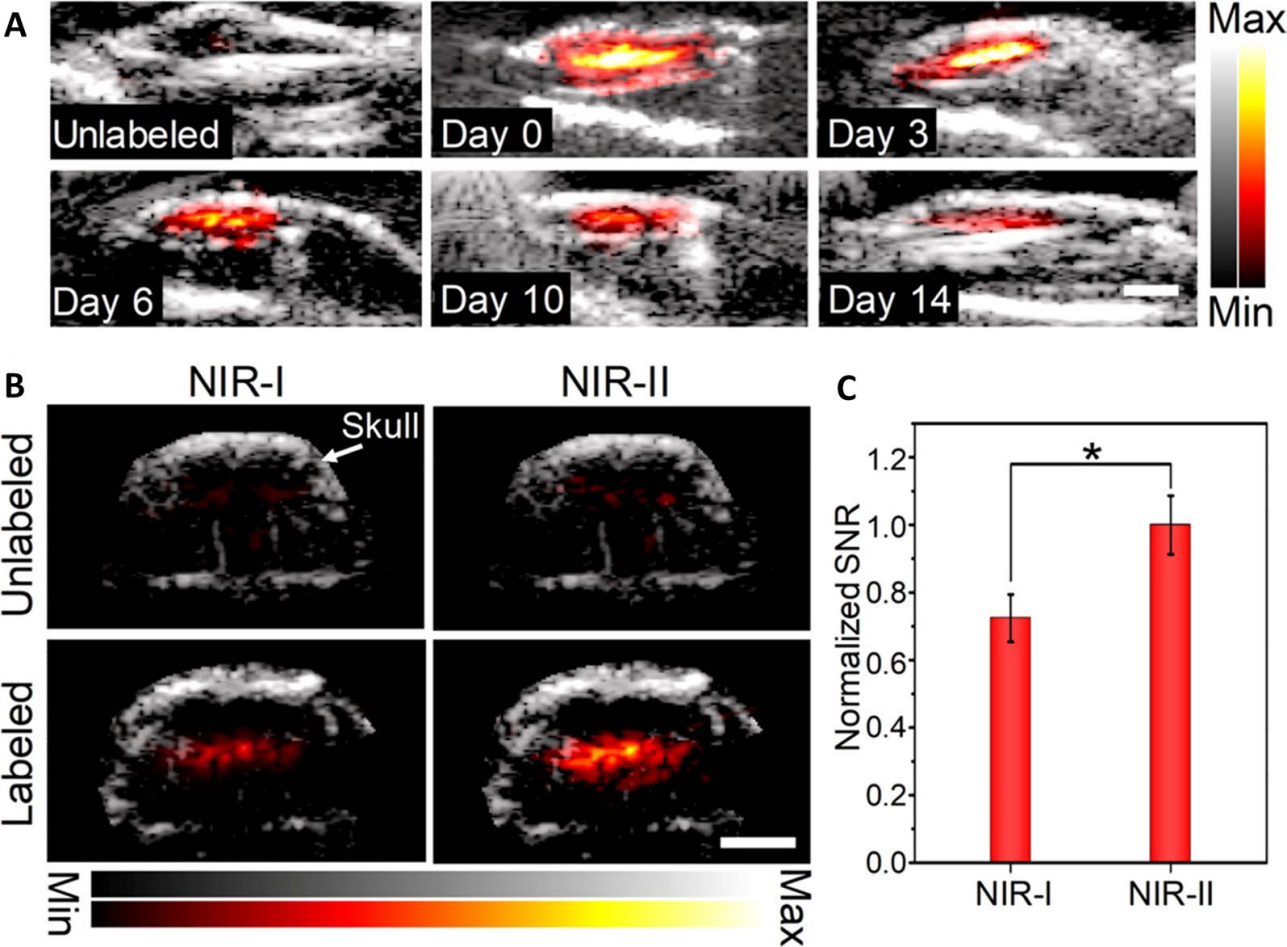
PA imaging in vivo. **A** Merged ultrasound (US, gray scale) and PA (red-yellow scale) images of unlabeled or OSPNs+-labeled hMSCs. Images of OSPNs+-labeled cells were acquired serially at different post-injection time (days 0, 3, 6, 10, and 14). PA signals were recorded at 1064 nm (energy density: 5 mJ/cm^2^). Scale bar: 3 mm. **B** Combined US and PA imaging of unlabeled or OSPNs+-labeled hMSCs injected into the brain of nude mice using NIR-I (860 nm) or NIR-II (1064 nm) light excitation (energy density: 10 mJ/cm^2^). Arrow indicates the skull position. Scale bar: 3 mm. **C** Normalized PA SNR of OSPNs+-labeled hMSCs implanted into mice brain under NIR-I (860 nm) or NIR-II (1064 nm) light excitation (*p < 0.05). The image was reproduced from article published by Yin, Pu, Wang, Bian and co-workers in ACS Nano journal in the year 2018 [97]

Semiconductive polymeric nanoparticles comprise the major part of organic nanoparticles that possess PA and fluorescence properties because they have NIR absorption property. In addition to their exceptional photostability and biocompatibility, semiconducting polymers possess distinct advantages over the other nanoparticles in PA applications [90]. Successful detection of reactive oxygen species (ROS) using polymeric nanoparticles as nanoprobes has demonstrated that, these nanoparticles can be an excellent tool for the diagnosis of disease conditions such as tumor metastasis, cardiovascular diseases, diabetes, obesity and etc., that involves ROS guided/influenced pathophysiological events [87]. Similarly, quantum dots represents another group of multifunctional nanoparticles with significant potential in photothermal therapy (PTT) and has made them a promising candidate to be a PA contrast agent [90]. Perfluorocarbon (PFC) are microbubble contrast agents, that showed great potential in ultrasound/PA based therapies and stimuli responsive drug delivery, when they were coupled with plasmonic nanoparticles [96]. Diketopyrrolopyrrole (DPP) based polymeric nanoparticles and their derivatives were aided in tumor imaging with more than fivefold of the background signal at 2 h of the nanoparticle injection [87, 97]. Benzothiadiazole moiety amplified the PA signal provided by the nanoparticles by twofolds [98]. Apart from imaging application for diagnosis, PA image guided photothermal/photodynamic therapy (PTT/PDT) was also achieved using semiconducting polymeric nanoparticles (Fig. 4) [97, 99].

### Organic dyes-based nanoparticles

**Fig. 5:**
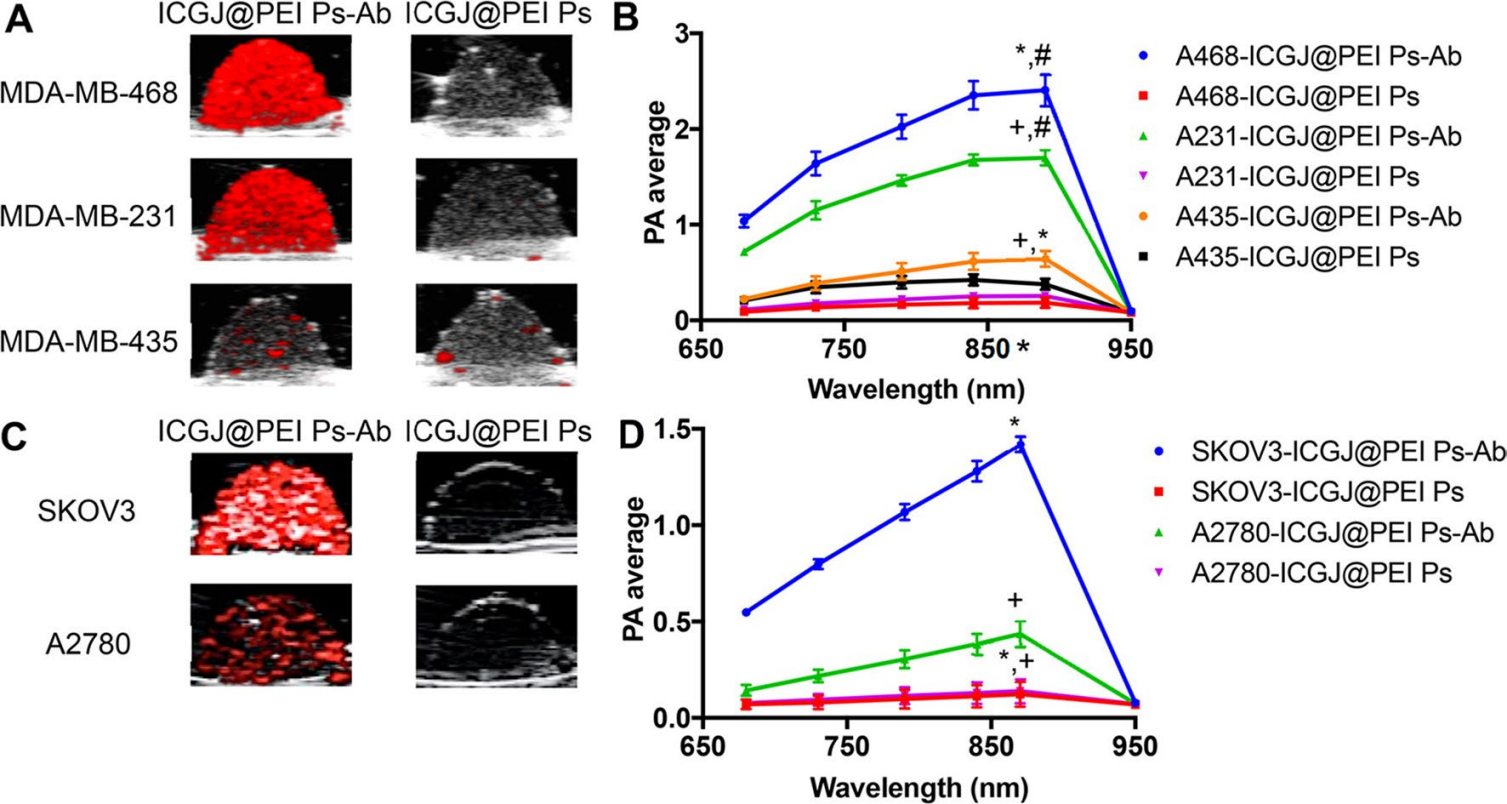
PAI of breast and ovarian cancer cells. **A** Cross-sectional PA images at 890 nm excitation overlaid B-mode ultrasound of gelatin phantoms containing inclusions of breast cancer cells MDA-MB-468, MDA-MB-231, and MDA-MB-435 labeled with either EGFR-targeted (ICGJ@PEI Ps-Ab) or nontargeted (ICGJ@PEI Ps) polymersomes and **B** corresponding PA spectra that are based on volumetric PA signal analyses. **C** Cross-sectional PA images overlaid B-mode ultrasound of gelatin phantoms containing inclusions of ovarian cancer cells SKOV3 and A2780 labeled with either EGFR-targeted (ICGJ@PEI Ps-Ab) or nontargeted (ICGJ@PEI Ps) polymersomes and and **B** corresponding PA spectra that are based on volumetric PA signal analyses.(each value shows mean ± SD based on n = 3 samples). The image was reproduced from article published by Changalvaie, Johnston and co-workers in ACS Applied Materials and Interfaces journal in the year 2019 [104]

Organic dyes are continuously being explored for its PA contrast property as they exhibit equivalent PA enhancement compared to polymeric nanoparticles an have appreciable biocompatibility. They are relatively cheaper compared to polymeric nanoparticles [100]. ICG dye-based nanoparticles with its high NIR absorption property showed best photoacoustic effect and its application in PA imaging was approved by FDA (Tricarbocyanine fluorophore) [101]. ICG has shown its potential in PA imaging for diagnostic applications and PA imaging guided PDT/PTT for cancer theranostics. Several nanoformulations of ICG has been appreciated for its applications in multimodal imaging including magnetic resonance imaging (MRI) (Fig. 5) [102–104]. Porphyrin based nanoparticles possess excellent absorption of Soret and Q bands and this aids in PDT [105]. Detection of uranium by PA imaging was the first instance of porphyrinoid nanoparticles application in PA imaging [106]. Conjugation with phospholipid makes porphyrin nanoparticles effective in PA imaging and also reported as an integral part of photonic microbubble, that acts as both US and PA contrast agent [105].

**Fig. 6:**
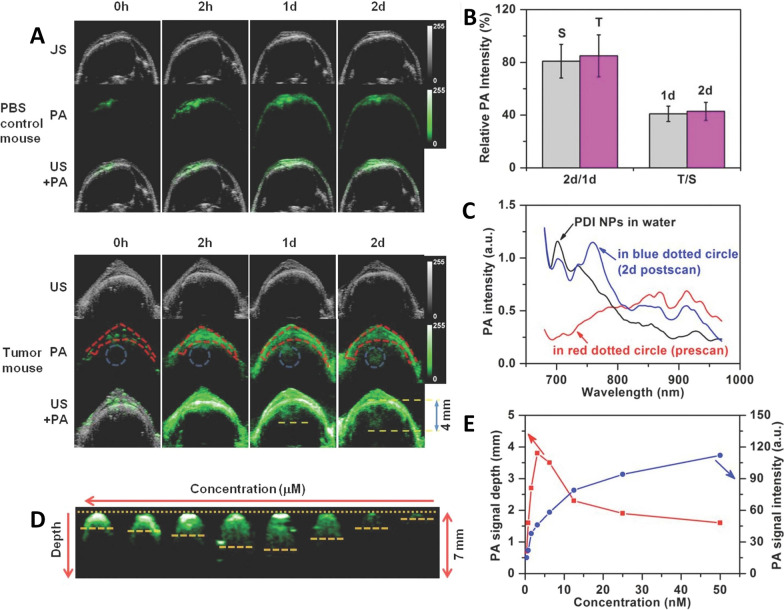
**A** The US (grey), PA (green), and their overlay coronal sections of brain of control model (top) and tumor model (bottom) before and after tail vein injection of 250 µL of 250 nM PDI NPs. **B** The relative PA signal changes of the skull region (S, in red dotted circle of **A**) and tumor region (T, in blue dotted circle of **A** at 2 d compared with that at 1 d, respectively, and that of T/S at 1 d and 2 d. **C** PA spectra of PDI NPs in aqueous solution (black line), the region in red dotted circle of **A** before injection of NPs (red line) and tumor region of **A** after 2 d injection of NPs (blue line). **D** The PA coronal sections of PDI NPs with different concentrations (from 50 to 0.390625 nM) in agarose gel (in vivo phantom). **E** The plots of PA intensity–NP concentration and PA detectable depth–NP concentration relationships. The image was reproduced from article published by Fan, Cheng and co-workers in Advanced Materials journal in the year 2015 [111]

Melanin is a naturally occurring pigment and their strong NIR absorption was utilized in PA imaging. They also possess excellent physiological stability and is considered as a good chelating agent [107]. Multimodal imaging was successful with melanin-based nanoparticles, with their metal ion (Fe^3+^ and Cu^2+^) chelating property in tumor detection. Integrin (α_v_β_3_) was targeted by RGD coupled melanin nanoparticles and U87MG tumor in mice was detected with enhanced PA signal 4 h post injection [108]. Alteration of pH and oxidation state of the microenvironment has aided melanin nanoparticles in imaging the tumor vasculature and microcirculation [109]. HER2 tumor was also detected by PA imaging with the help of melanin nanoparticles and enhancement in the PA signal was observed with TNF-α administration [110]. Perylene diimide (PDI) and squaraine (SQ) dyes based nano contrast agents are also successful in PA imaging and tumor diagnosis. PDI based PA imaging has successfully detected brain tumor facilitated by the excellent absorption characteristics of PDI around 700 nm. Additionally disruption of BBB has led to enhanced permeability of PDI nanoparticles, which helps in visualizing deeper regions in the tissues (Fig. 6) [111, 112]. SQ were utilized as PA nanocontrast agent in aggregated form rather than a single molecule. Dual imaging (NIR/PA) of tumor was successfully carried out with nano-SQ. Encapsulation of SQ couples with halogenated dicyanovinyl derivatives in liposomes showed reduction of fluorescence and enhancement of PA signals upto tenfolds [113]. Liver imaging was successful done with the help of SQ-albumin based nanocomplexes, which has very good NIR absorption property [7].

### Nanocomposites

**Fig. 7:**
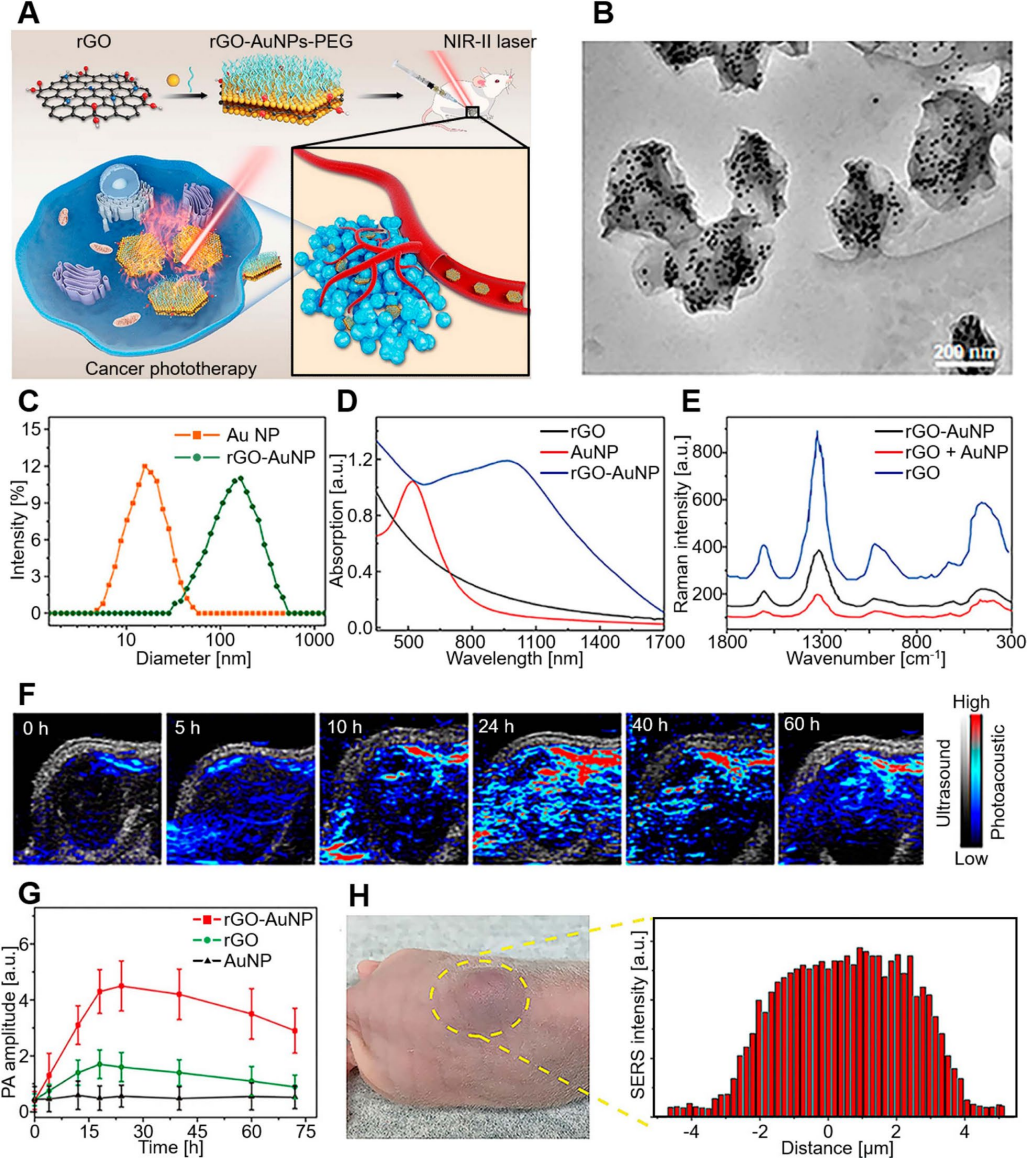
**A** Schematic diagram of rGO–AuNP–PEG synthesis and PAI-guided PTT for cancer ablation in the NIR-II window. **B** TEM images, **C** hydrodynamic distribution, **D** UV–vis spectra, and **E** SERS spectra of rGO–AuNPs. **F** PA images and **G** PA amplitude after 1250 nm laser irradiation. **H** In vivo SERS signal of the tumor and its surrounding tissues to determine the boundary between tumor and normal tissue. The image was reproduced from article published by Kang, Eom, Kim, Han and co-workers in Biomedicines journal in the year 2022 [116] and Wang, Wang and co-workers in Frontiers in Bioengineering and Biotechnology in the year 2020 [117]

Carbon based nanomaterials are considered commonly for variety of biological applications because of its excellent optical properties and simple fabrication methods. PAI properties of carbon-based nanomaterials has given liberty to the scientists to develop carbon-based nanomaterials/composites that can be utilized for multimodal theranostic applications [114, 115]. Graphene-based nanocomposites are widely used for many applications and with respect to PAI, the NIR absorption property has given structural stability, photothermal and PA contrast properties to the graphene oxide (GO) based nanocomposite (Fig. 7) [116, 117]. GO decorated with iron oxide nanoparticles encapsulated in polyethylene glycol, are used for multimodal imaging with superparamagnetic property of IONPs rendering contrast enhancement for MRI and PET and GO enhances the PA signals [118]. PEGylation and gold nanoparticle conjugation has achieved more than fivefold increase in NIR absorption of GO nanoparticles [119]. BSA conjugated with reduced GO nanoparticles were also used in PA imaging and it exhibited minimal toxicity [120]. Similarly single walled carbon nanotubes (SW-CNTs) were also known for its NIR absorption property and have been extensively utilized for cancer therapy, particularly in PTT owing to their effective light-heat conversion. SW-CNTs combining with RGD peptides showed excellent systemic stability and showed significantly amplified PA signals during tumor detection in vivo [121]. Dye conjugated SW-CNTs or GO were also reported to be a potential PA contrast agent and nanomedicine for cancer through PDT/PTT [93]. Significant exploration on carbon-based nanomaterials and nanocomposites led to development of cone shaped single walled carbon nanohorns and nanodots that are examined for its diagnostic and therapeutic applications. Nanohorns were utilized for PA imaging guided PTT, while nanodots were developed as nanocomposites with contrast enhancing organic dyes for diagnostic purpose and with chemotherapeutic agents for PDT or PTT applications [122, 123]. Nanocarbon with gold-based nanomaterials, silica-based nanoparticles and other inorganic sources like Ni, Pd or Cu based nanocomposites are examples of hybrid materials, successful in many bioimaging applications including PA imaging [115].

## Targeted delivery of nanoparticles for photoacoustic applications

Nanomaterials are potential tool for diagnostic and therapeutic strategies with great success in several disease management processes. On the other hand, experts are involved in constant exploration on addressing the issues faced during the nano diagnostics/therapeutic strategies. Major influencing factors of a successful nanomedicine are, physico-chemical characteristics of the material, physiochemical responsiveness (pH, Temperature, redox status and etc.), pharmaceutical parameters (biodistribution, plasma concentration, drug release pattern and etc.), enhanced permeation/retention effect (EPR), cellular uptake, therapeutic efficacy and toxicological profile [124, 125]. However, the route of administration is still remained underappreciated compared to factors such as safety, biodistribution, that are focused during fabrication of nanomedicine. Administration route is a vital factor in success of nanotheranostic agents because, the obstacles that formulations encounter in each route varies and has its own potential against the stability and efficacy of the material. Intravenous route is the commonly preferred route for nanomaterial which showed significant therapeutic effect on cancer and certain vascular complications, but the excess accumulation in the liver and spleen poses a possibility of inflammation and long-term toxicity. Oral administration is a preferred mainly in a clinical set up because of its safety and convenience in administration, however, the materials must undergo several barriers at the mouth cavity, mucus, and GI tract. The barriers can be evaded by local administration at the region of interest to aid in faster and organ specific delivery of the material. This route can be exploited for superficial tissues and topical application and not suitable for the deep tissues targeting [126, 127].

Nanomaterials with the specific physical property are employed either directly in respective imaging modalities or transformed the contrast agents into a nano form. This aids in acquiring better images and provide specificity in the diagnosis [75]. Several nanocontrast agents were developed and successfully employed in clinics for MRI, CT, US and similar imaging techniques, for an improved diagnosis for various diseases [83]. In the past decade, several research studies are carried out to develop contrast agents to support PAI and related techniques as they provide significant insight into the disease pathogenesis, especially cancer diagnosis and PAI guided cancer therapy [90, 128]. Nanoparticle targeting strategy for PAI can be classified as active and passive targeting. The phenomenon where nanocontrast agents require an external stimulus for accumulation at the region of interest or depend on the enhancement of the permeation (EPR effect) is called passive targeting, while targeting them by enzymatic release at the tumor microenvironment, receptor mediated endocytosis or molecular targeting strategies were collectively termed as active targeting techniques [16, 126]. Pharmacokinetic profile of the PAI contrast agents were explored and efforts are taken to optimize the formulation which are stable in the physiological system and are less toxic. On the other hand, formulations of PAI contrast agents were also constantly being improvised for site specific targeting, enhanced bioavailability and less systemic toxicity. With respect to cancer diagnosis, therapy guided by PAI, visualizing the tumor microenvironment and etc., intratumoral injection or locoregional administration has been a common approach. However, differentiating the benign from metastatic lesion or staging of the disease can be possible with imaging performed at the molecular/cellular levels. In this regard, the PAI was reasonably successful compared to other imaging methods and based on the influence of lymphatic system on cancer pathogenesis, which is the commonly selected region of interest for staging and differentiation of cancer progression [121].

## Challenges for photoacoustic imaging

Success of PAI is an inevitable consequence of successful ongoing research studies and its improving potential applications owing to the technological developments. However, there are many notable challenges reported during its translation to the clinical set up such as adverse effects of laser, requirement of PA contrast agent when the natural PA effect was not obtained and certain other challenges, which we will discuss in this section [129]. Observation of blood vessel characteristic and measurement of blood oxygenation levels has made PAI a most sought-after clinical imaging tool [130]. With the help of natural PA contrast enhancing agents, angiogenesis in cancer microenvironment, atherosclerotic plaques, vascular tissue compositions can be visualized [24]. However, this may not be sufficient for diagnosis of diseases which has complex pathological stages during progression. Inability to obtain signals from deep tissue pose a serious challenging for the experts during PA imaging [16]. On the other hand, laser exposure was always set below the maximum permissible exposure (MPE) and this particular parameter has to be considered during optimization of the PA conditions. However, better signals were obtained when the laser intensity was increased above the MPE limit which may cause skin irritation and other toxic responses [131]. Several studies have been conducted to address these issues and researchers concentrated on development of contrast enhancing agents for improving the PA signal. Including PAI, many imaging methods rely on nanoparticles-based contrast agents for better clinical presentation with improved image quality. However, adverse reactions due to the injected contrast agent are still another major hurdle to cross in PAI based diagnostic process [92, 132]. PA contrast agents based on metallic nanoparticles, though have provided significant assistance in enhancing the PA signals, it showed high bioaccumulation and long-term toxicity [93]. Smaller nanoparticles though showed less systemic interaction, it had very less circulation time, which was not enough for the imaging [133]. Biodegradable nanoclusters showed little success with respect to reduced toxicity and increased cellular uptake, but their fate inside the cell due to change in pH to 5 from 7 (outside the cell) has resulted in small gold nanoparticles and they were eliminated easily once the polymeric segment of the nanoparticles were degraded [134]. Recently the polymeric nanoparticles were identified for its NIR properties and are under continuous exploration for development of a stable, non-toxic PA nanocontrast agent [135]. Contrast enhancing agents have also addressed certain other technical difficulties like providing “wet” contact environment to the bridge the tissue of interest and transducer. Vital clinical decisions can be made based on the contrast enhanced PAI when they provided molecular intracellular information on expression levels of certain important biomarkers/enzymes [12]. Image obtained from a photoacoustic system is a product of the optical absorption and light fluence from the region of interest (ROI). However, the acoustic signal reception from the ROI is a very challenging task resulting in limited image view point and this increases the chance of artifact. Combining with the difficulty of decreasing signal to noise ratio in the deep-seated tissues, the clinical translation of PAI imaging has become very hard to translate in the clinical scenario. The ROI at a greater depth has poor PAI signals because, when tissue depth increases the ROI will be surrounded by varied tissue structure [128, 136]. Speed of sound and the Grüneisen coefficient of various tissues remains fairly consistence, however, their accuracy is observed to reduce with increase in depth causing interference from the surrounding tissue structure. This greatly affects the quantitative analysis of the output images [12]. Temporal resolution of PAI systems is another important factor yet to be addressed to improve image outputs and experts are focusing on development of real time PA imaging methods to address temporal resolution and related challenges [137, 138].

In case of cancer diagnosis, PAI has developed significantly, but the basic challenges described above had interfered the path of PAI to the clinical translation. Diagnosis of tumor from deep tissue and further differentiating the cancer cells and staging them is still a great challenge. As these data will be instrumental in arriving at a clinical decisions that are critical for the disease management, and the data lacks clinical value without a definitive observation. However, PAI with its ability to delineate the blood flow characteristics, comprehensive information about the tumor microenvironment can be extracted, as angiogenesis around the tumor region will be distinct enough to clearly differentiate the margin from the healthy tissue under PA imaging systems [136]. Since, tumor microenvironment has poorly developed lymphatic system, PAI of the lymphatic system helps in delineating the tumor margin. Further, lymph nodes and lymphatic circulation play a pivotal role in cancer progression and metastasis. Hence, lymphatic imaging can also help in prognosis of tumor recurrence after surgery and therapy [139, 140].

PA imaging of lymphatic systems architecture and the dynamics of the lymphatic circulation has facilitated the timely and accurate diagnosis of lymphatic disorders, conditions that can has significant impact on lymphatic physiology and diseases whose pathogenesis involves lymphatic system [141]. Among several disease conditions that are influenced by lymphatic system, cancer is a prominent example as the anatomy of lymphatic system aids in cancer cell mobility and apart from detection in the systemic blood circulation, circulating tumor cells were also identified in the lymphatic circulation [142]. Hence, early signs of cancer metastasis can be identified through lymphatic imaging. PAI has shown significant promise in cancer diagnosis recently especially through lymph node imaging. However, lack of endogenous PA contrast enhancing molecules in the lymphatic circulation, an external contrast agent, generally the nanoform of PA signal amplifying molecules/dyes should be administered. As discussed earlier regarding the challenges and obstacles in site targeted delivery of nanomaterials, researchers have been exploring the various means for lymphatic delivery of PA contrast agents [9].

## Lymphatic system and cancer progression

### Anatomy and physiology of lymphatic system

Lymphatic system bridges the tissues to blood and acts as an important check point for immune system and their response to several physiological and pathological processes. It plays a key influence in lipid absorption, transportation of cells and fluids from the interstitial space back to the systemic circulation [143]. Lymphatic vessels drain the fluid to the lymph nodes and it is one way circulation that flows through spleen, thymus, tonsils and Peyer’s patch. Lymph drainage from tissue was aided by pressure gradient due to outward push of fluid by blood vessels or inwards suction created by pumping of lymphatic vessels. The collected fluid joins the lymphatic trunk which ultimately rejoin with the venous circulation through subclavian vein. The lymphatic endothelial cells form the thin-walled lymphatic vessels that can accommodate larger particles with size more than 100 nm from the circulation after phagocytosis in the interstitial space and reach lymph nodes. Lymphatic pumping plays an active role during fluid homeostasis. The lymphatic pumping, unlike blood circulation which is single pumping mechanism, is a distributed system of pumping [144]. Some well understood mechanisms of lymphatic pumping are calcium-based dynamics helping in lymphatic muscle contraction [145], mechanical activation [146] and biochemical modulation induced by endothelial relaxing factors such as nitric oxide, glycocalyx components, adhesion molecules and etc. [147, 148]. As mentioned above the critical function of lymphatic system is to absorb and transport dietary lipids apart from being a carrier fluid and immune cells [144].

### Lymphatic system and related disorders

Dysfunctional lymphatic circulation has been reported to cause lymphedema (primary and secondary) and accelerate the progression of obesity, fibrosis in several organs, autoimmune diseases, cardiovascular complications, inflammatory bowel disease, ocular diseases, neurological and neurodegenerative disorders. Swelling in different parts of the body is a result of abnormal fluid accumulation due to impaired lymphatic circulation [149]. Genetic predisposition of lymphatic dysfunction results in primary lymphedema such as genes pertaining to lymphagiogenesis (VEGFR3, FOXC2, SOX18, ITGA9 and etc.) are the influencing factor of primary lymphedema in defective form [150]. On the other hand, secondary lymphedema is caused as a consequence of surgery (especially cancer resection surgery), infection (filariasis) and radiation that damages the lymphatic vessel. Auto immune diseases such as Rheumatoid arthritis or systemic sclerosis had a vague correlation with defective lymphatic circulation [151]. Most reported causes of the secondary lymphedema are damage in the lymphatic vasculature during tumor resection along with lymph nodes and during radiation therapy for cancer [152]. Metastatic tumor cells take the lymphatic route to affect distant organs and is particularly successful when the immune regulation function of lymphatic system is compromised. Apart from the pathological conditions caused due to malfunction of lymphatic system, their response to several diseases conditions has gained interest of the experts to target the lymphatics for improved stability, delivery and efficacy of the drug intended against disease of interest.

**Fig. 8:**
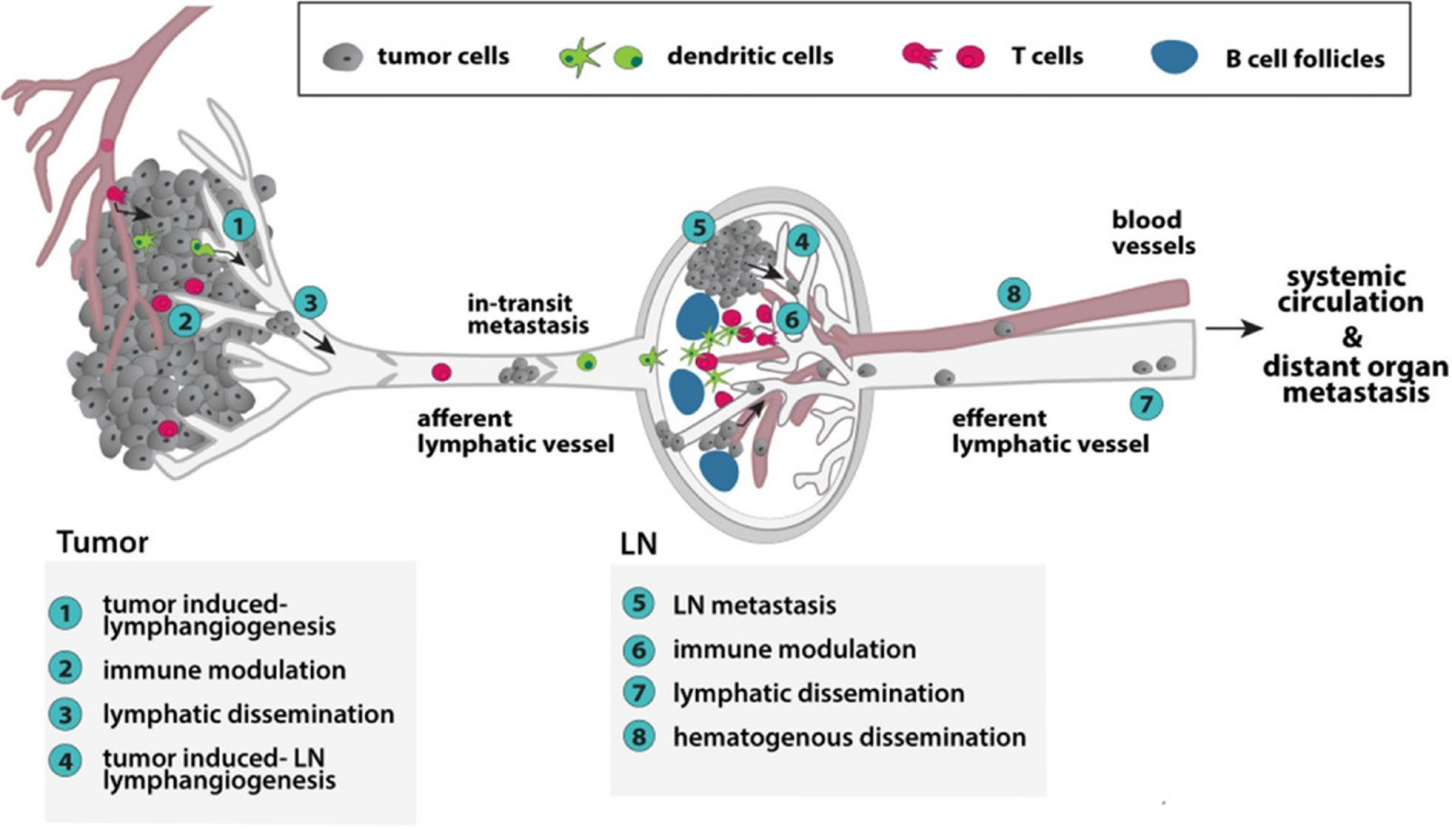
Schematic illustration of the principal routes of lymphatic metastasis and roles of lymphatic vessels in tumor progression. The figure was reused from the review article published in Medicinal Research Review by Rezzola, Halin, Ronca and co-workers in 2020 [148]

### Role of lymphatic circulation in cancer progression

Circulating tumor cells are commonly observed in the lymph nodes which indicates the metastases of solid tumors and prognosis becomes poor when the cancer cells drain into the lymph node. Though this phenomenon is under significant scrutiny, the patients were generally advised for systemic therapy. It is believed that lymph node metastases should be considered as the priority before focusing on other organs. However, this may vary according to individuals’ circumstances (Fig. 8). Lymphatic trafficking of cancer cells is a multistep process involving attraction, entry, and lymph node penetration. Lymphatic circulation is constrained up to peritumor margin and lack of intra tumoral lymphatic vessels leads to interstitial fluid pressure and cancer cell drainage into the lymph nodes. Tumor derived VEGF promotes contraction of lymphatic collecting vessels and this helps in tumor cells distribution throughout the system. Cancer cells proliferate in the lymph nodes and this causes resistance in the lymphatic vessels causing collateral re-routing of the lymph flow, around the tumor mass [148].

Premetastatic niche is an important factor that regulates the systemic circulation of the tumor cells, successful colonization of the tumor cells at distant organs. Lymph node at premetastatic stage show several features like lymphangiogenesis, lymph flow, remodeling of venules, myeloid cells infiltration and reduction of lymphocyte number [153]. These events were regulated by molecules that are secreted by primary tumor at the sentinel lymph node. Lymphangiogenesis is triggered by VEGF (A and C), integrin α_4_β_1_ and erythropoietin which correlates with metastasis [143]. High endothelial venules (HEVs) are another important part of many cellular types, that helps in recruitment of inactive lymphocytes and anti-tumor immunity. But they can be impaired during tumor cell translocation into the lymph nodes. Apart from this, the oxygen and nutrient supply to the lesions in lymph nodes [154]. Dysregulation of certain signaling molecules like growth factors, cytokines and prostaglandins expressed through different cell types, have significant role in tumor draining to lymph nodes and creating the premetastatic niche [153].

Survival of cancer cells after their entry into the lymphatic circulation is an important step, as they are likely to experience hypoxia and cancer cells achieve the lymph node invasion by utilizing the oxygen from the native vasculature [155]. The signaling molecules like CXCR4, CCR7 and several other chemokines and cytokines were responsible for adaptive immune processes in the lymph nodes. However strong evidences were reported on cancer cells utilizing these signaling molecules and pathways for lymph node invasion [156]. This process also requires the ability of cancer cells to escape the immune response, which is performed through modulation of several immune signaling processes like reduction of number of effector T cells by increased facilitation of inhibitory T (regs) cells that could suppresses T cell proliferation [157], modulation of lymphatic endothelial cells to influence the presentation of foreign and self-antigens, leading to cancer cell survival [158] and also hindrance with the maturation of dendritic cells [159]. HEVs are also reported for their ability to impair immune cell entry into the lymphatic circulation [154]. Additionally, B cells also involve in affecting the lymphatic ability to control immune response and aid in lymph node metastasis [160]. These findings related to role of lymph node in cancer growth and its metastasis have highlighted the fact that, anticancer drugs targeting primary tumor will be less effective against metastasis. Hence, drug efficacy will be enhanced only if the all the tumor microenvironments (including lymph node metastasis) that are involved in cancer progression, was taken into consideration during drug development [161]. Small molecule drugs, immunomodulatory compounds and other anti-cancer agents were targeted towards lymph node by subcutaneous administration. Drugs and other therapeutic agents were administered in the form of lipid nanoparticles has shown significant drug efficacy. Similarly, the adjuvant in nanoform elicits the immune response against the tumor antigens in the lymphatic circulation [162].

## Developments and challenges in clinical translation of lymphatic imaging using photoacoustic systems for cancer management

Early and accurate diagnosis is an important aspect in a successful cancer management process and this is achieved by instrumentation and diagnostic methodologies that can precisely decipher the cancer progression stages and metastasis in a shorter duration compared to the other methodologies [163]. PAI holds significant edge over other imaging methods with respect to lymphatic imaging due its deeper penetrability and on par resolution with certain imaging methods like MRI, CT and etc. However, there are many hurdles to cross before PAI becomes a routine diagnostic imaging method for cancer and other similar, complex diseases pertaining to lymphatic circulation [9]. Biopsy sampling of SLN is still considered as the most reliable methodology for diagnosis of metastatic lesions. However, palpability of sentinel lymph node (SLN) is a major challenge and thus the biopsy sampling process require guidance of radioactive materials or surgery. Exploration for better methodology to overcome the invasiveness of surgery and radioactive substances for SLN biopsy and several researchers exhibited that PAI as one of the reliable and better alternatives. Several in vivo studies were conducted with photoacoustic systems and has achieved significant success in lymphatic imaging-based diagnosis of cancer progressions that are comparable to histological observations with minimal invasiveness [12].

PAI and pertaining modalities have shown great success in lymphatic mapping, and aided in analyzing the status of lymphatic circulation during the progression of cancer, especially at the metastatic state. PAM and PAT are the currently successful PA-based systems in vivo, and are enroute to clinical imaging sector [12]. Administration of methylene blue (MB) dye was reported to aid in tracking the SLN in rats. PAM imaging after intradermal injection of MB in rats helped in SLN mapping with an optical resolution of 500 μm for almost 200 mm depth [9]. However, following the emergence of metal-based nanomaterials as PA contrast agents, gold nanocages and gold-coated carbon nanotubes showed greater penetrability of PAM and the nanoparticles are reported to arrive lymphatic system rapidly accumulate in the lymph nodes, which helps in receiving stronger PA signals. These studies have showed the possibility of tracking the lymphatic circulation and anomalies due to cancer or any other metabolic disorders can be accurately diagnosed for arriving at a clear-cut therapeutic strategy [36, 134]. PAI for diagnosis of cancer and its progression at clinical level was experimented with ICG dye that can aid in fluorescence and PA imaging together (dual mode) for SLN mapping to stage the cancer progression along with evaluation metastatic potential of the tumor in patients using a handheld PA imaging system that locates the SLN and tumor tissue for biopsy sampling. Fluorescence imaging provided excellent temporal resolution, while PAI aided in improvement of spatial resolution [164]. Similarly, MB dye helped in biopsy needle guidance for SLN sampling using PAI, while MB after radiolabeling has facilitated SLN mapping by single photon emission computed tomography [17]. PAI has also been explored for its ability in ex vivo SLN assessment, which can be faster and more accurate compared to histological and immunohistochemical analysis that could take weeks for diagnosis and by then the metastasis might have progressed significantly leading to rely only upon lymphadenectomy for the patients. MSOT is also an effective methodology in lymphatic system mediated cancer diagnosis with a very good sensitivity (100%) and specificity of more than 60%. SLN at a depth of 50 mm was successfully analyzed in 20 patients reported with melanoma using ICG as an exogenous contrast agent, along with that spectral information of melanin was obtained to identify the metastatic status of SLN [12].

Collectively the potential of PAI to be an alternative to current clinical imaging methods was clearly demonstrated by these studies. Though PA based methods show high specificity and sensitivity, the false positive results are still a concern. Apart from this, several challenges in PAI are raised that can halt its routine usage in clinics. Some of the important challenges in imaging processes that hinder the clinical translation of PA imaging includes, development of suitable contrast agent since lymphatic system does not contain endogenous contrast enhancing molecules, lateral resolution is not on par with fluorescence imaging or any other radiological imaging systems, motion artifacts, data processing and interpretation [136]. With respect to instrumentation of the PA imaging systems difficulties in integration with the clinical work flow by aiding patients with smooth imaging process is an important concern. Regulatory approval is another major hurdle to be crossed by any imaging method and pertaining drugs agents including PAI contrast agents. Standardization of the instrument and well-trained operating personnel are vital requirement for the successful PA imaging. Constant developments in the research domain provide a clear forecast on PAI systems which can be commercially available with more affordable cost with less side effects to the patients [165].

## Nanotheranostic agent based photoacoustic imaging of lymphatic system for cancer diagnosis and guided therapy

Development and success of nanomaterials have shown significant improvement in lymphatic targeting and cancer therapy [166]. Therapeutic targeting and efficacy of the nanomaterials were significantly improved by an external stimuli like heat, sound or light that can aid in activation of drug molecules in nanoparticles, targeting of specific organ or cell types, accumulation of drug molecules at the site of target and facilitation of drug entry into the cell by changing the membrane dynamics etc. External physical stimuli thus help in enhancing the diagnostic and therapeutic efficacy of the nanoformulation [167]. Among the lymphatic imaging methods, PAI has shown significant success with the ability of US to be detected from deep tissues with resolution and has a high spatio-temporal resolution. The imaging method is non-invasive and it has less scattering effect on the images obtained [9, 141, 168]. Among the disadvantages PAI of lymphatic system absence of endogenous contrast enhancing agents like Hb for blood vessels imaging is still an unaddressed concern. However, the contrast agents can be engineered or manipulated to aid in drug delivery, targeting and therapeutic action [89, 169]. The potential of PAI in lymphatic imaging and the role of lymphatic system in cancer progression has shown path to the experts for encountering the challenges posed by metastasis during cancer management. Further, the ability of enhanced PAI theranostics using nanotechnology-based tools has grabbed significant attention in the past decade [168, 170].

Cancer detection was facilitated by several imaging technique that should be focused on differentiating the cancerous from benign lesion, identifying the level of cancer progression and finally identify the metastatic lesion. On the other hand, cancer therapeutics are of several types that targets multiple pathways influencing cancer progression [163]. Lymphatic systems are the key modulator for progression of several types of cancer in to metastatic state. Hence, lymph nodes and pertaining structures were imaged as well as targeted in cancer management [171]. Among the diagnostic imaging methods PAI has shown significant development and recently research progress is towards PAI based theranostic applications for cancer [12]. PAI enhancing nanocontrast agents were successfully developed as well as formulations of nanomedicine for lymph node targeted delivery were also being developed. Apart from this, recent research studies are focused on development of nanomedicines with PAI contrast enhancing properties or coupling the therapeutic nanomaterials with PAI contrast agents [95]. In this section, we will focus on nanotheranostic materials that can target lymphatic system to influence key stages of cancer management.

### Differentiating non-cancerous and cancerous lesions

**Fig. 9:**
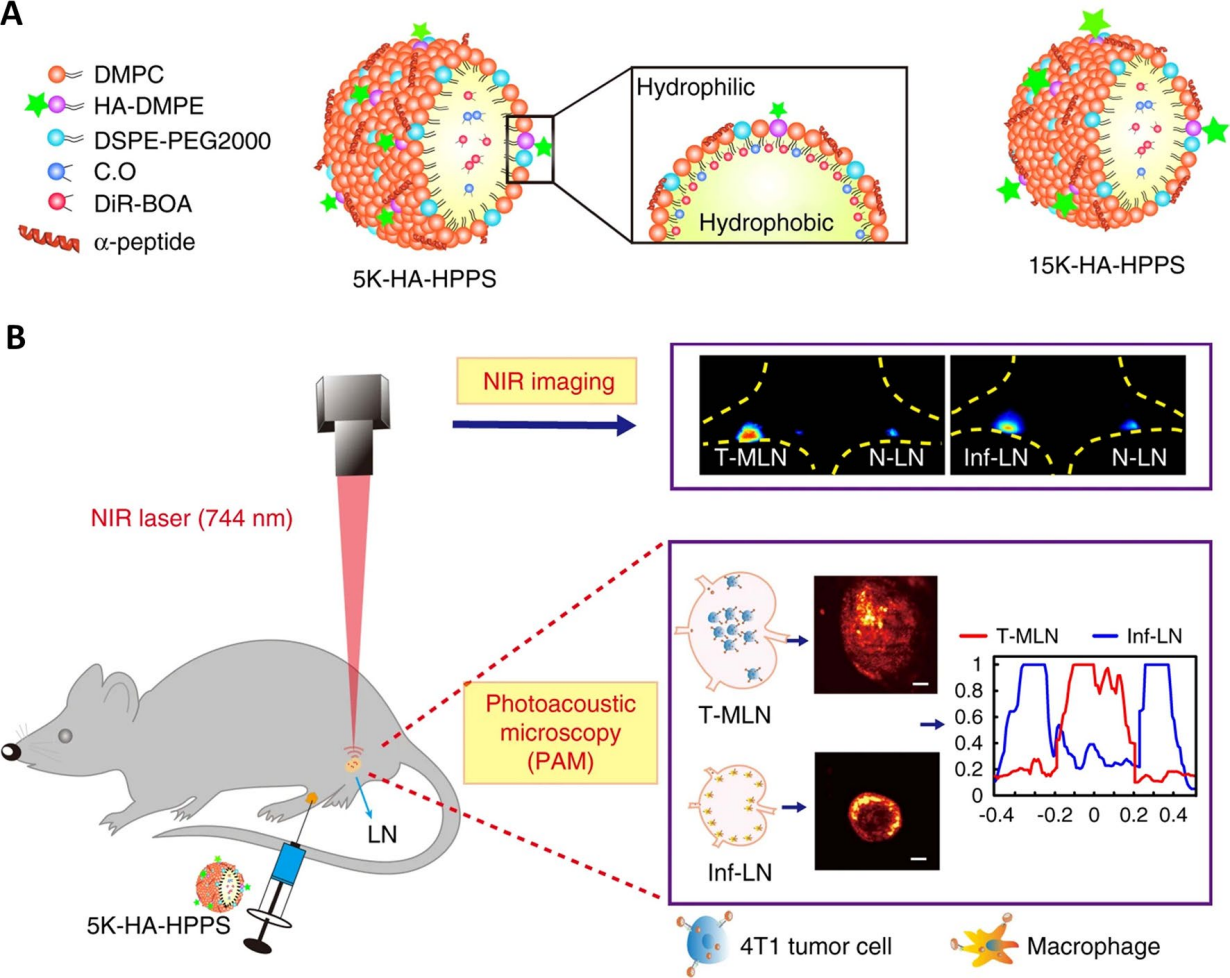
Design of dual-modality HA-HPPS nanoparticles for mapping sentinel lymph nodes in breast cancer. **A** Components and structures of the CD44 and SR-B1 dual-targeting HA-HPPS nanoparticles. **B** Dual-modality fluorescence and photoacoustic imaging of HA-HPPS in SLNs, which includes near-infra-red (NIR) fluorescence imaging for long-term monitoring of the accumulation and retention of HA-HPPS in SLNs and photoacoustic microscopy (PAM) for intraoperative determination of the metastatic status of SLNs in breast cancer. The figure was reused from the research article published in the journal Light: Science and Applications by Dai, Luo, Zhang and co-workers in 2020 [174]

Detection and demonstration of the cancerous lesions distinctly from benign and healthy tissues is a long standing challenge for the experts. Several imaging modalities are being explored continuously to overcome this issue [26]. PAI have been instrumental in this regard and a combination of imaging techniques along with PAI with or without multimodal theranostics has shown significant progress. Gold nanorods coated with silica has high affinity to tumor tissues and enhanced accumulation and corresponding PA signals were very helpful in specific detection of primary tumor. These rod-shaped metallic nanoparticles, Hb and oxygenated Hb were differentially observed by PA spectroscopy [172]. Multimodal imaging (MRI, CT, Raman spectroscopy and PA imaging) with metal-organic frameworks (MOF) were successful in differential imaging of glioblastoma through PA signals obtained from accumulated nanocomposites [173]. Differentiation of thyroid lesions from the usual benign nodules is a difficult task for the clinical experts. Trimodal imaging of thyroid and lymph nodes with PAI along with US and fluorescence methods have shown significant improvement in imaging based differentiation of the cancerous and non-cancerous lesions. This was achieved by studying the O_2_ saturation, vascular structure and blood flow parameters [16]. Targeted nano contrast agents were successful in distinguishing the tumor tissues from health mammary tissues in breast cancer model. CD44v6 antibody conjugation was able to establish the tumor margin clearly and differentiates with amplified PA signal (18-folds) (Fig. 9) [174]. Gold nanoshells were injected systemically to observe the neo vasculature and molecular indicators of the angiogenesis in the tumor mass. Contrast agent targeting α_v_β_3_ integrins and other angiogenesis mediating receptors helps in angiogenesis provide significant information about the tumor mass and delineates from the health tissues [121, 175, 176]. A phenomenon called enhanced permeation and retention effect has provided significant aid in the cancerous and non-cancerous cells differentiation [129]. Primitive vascular architecture of the tumor mass results in deposition of the nanoparticles in the tumor and this enhances the PA signals which is better than the signals from endogenous contrast agent like hemoglobin [134, 177]. Apart from this, PA was also helpful in intraoperative imaging of tumor for clear delineation of tumor border for complete resection. Rectal lesions were removed surgically guided by PAI after differentiated from adenomatous polyps [60]. Differentiation of metastatic, inflamed, and healthy tissues in SLN was successfully achieved during surgery by HDL mimicking peptide phospholipid conjugated with hyaluronic acid was injected [174].

### Staging of cancer and detection of metastatic lesions

Sentinel lymph node biopsy is the common procedure in staging of breast cancer and sometimes other types of tumor like melanoma or other skin lesions. This is a conventional invasive surgical procedure and experts were looking for alternatives to make the biopsy technique non-invasive. Recently, focus was shifted to PAI in guiding the fine needle aspiration of biopsies and specific gene expression analyses might provide a key information about the stages and progression of cancer [178]. Identification of metastasis in the lymph nodes plays a vital role in diagnosis of cancer, staging the progression and possible treatment methodologies [179]. Gold nanorods have been very instrumental in PA based tumor diagnosis, specifically in identifying the metastatic lesions. Gold coated carbon nanotubes (single walled) were used to identify the SLN in the mesenteric region to understand the metastasis and also was successful in mapping the lymphatic circulation [180]. Lymphatic mapping helps in quantifying the disseminated tumor cells throughout the lymphatic circulation. Apart from delineating the tumor margin, gold nanocages were also used in mapping the sentinel lymph nodes [181]. Micro metastasis is very important condition during cancer progression that occurs at the lymph nodes [182, 183]. PA guided flowcytometry helps in assessment of the migrating tumor cells, through the PA contrast effect of gold nanorods, nanoshells and carbon nanotubes as specific cellular markers [180]. However, the micrometastasis of melanoma cells can be observed with intrinsic contrast enhancement provided by melanin [184, 185]. Identification of circulating tumor cells (CTCs) are very important with respect to cancer management [186]. Metallic gold nanoparticles were successful in marking the CTCs in blood from breast cancers and prostate cancers [173]. Gold coated SW-CNTs and magnetic nanoparticles were together helps in trapping the CTCs magnetically from breast cancer and then they were detected using PA imaging [187].

### PAI guided surgical resection

The delineation of tumor margin with the help of PAI has revolutionized the tumor diagnosis and this has provided significant support in tumor resection surgery in the form of intraoperative imaging [136]. During removal of metastatic melanoma from the SLN, the intraoperative PA images has guided the surgical process and are comparable with the histopathological observations before the procedure. However, the discrimination of signals from blood and melanin was differentiated with reference PA spectra obtained prior to the surgery without the contrast agents. Then with the help of a set of algorithms, the ex vivo spectra of the lymph node were analyzed and the tumor margin was delineated [71, 188]. Hb and its respective endogenous contrast property plays vital role in PAI based therapeutics. Removal of metastatic tumor in the lymph node from oral cancer was successfully carried out with the help of the differential absorption between the oxygenated states of Hb. Further, the information about the extent of metastasis was also obtained from the PAI images which aided in obtaining optimal decision on proceeding with surgery [106, 189].

**Fig. 10:**
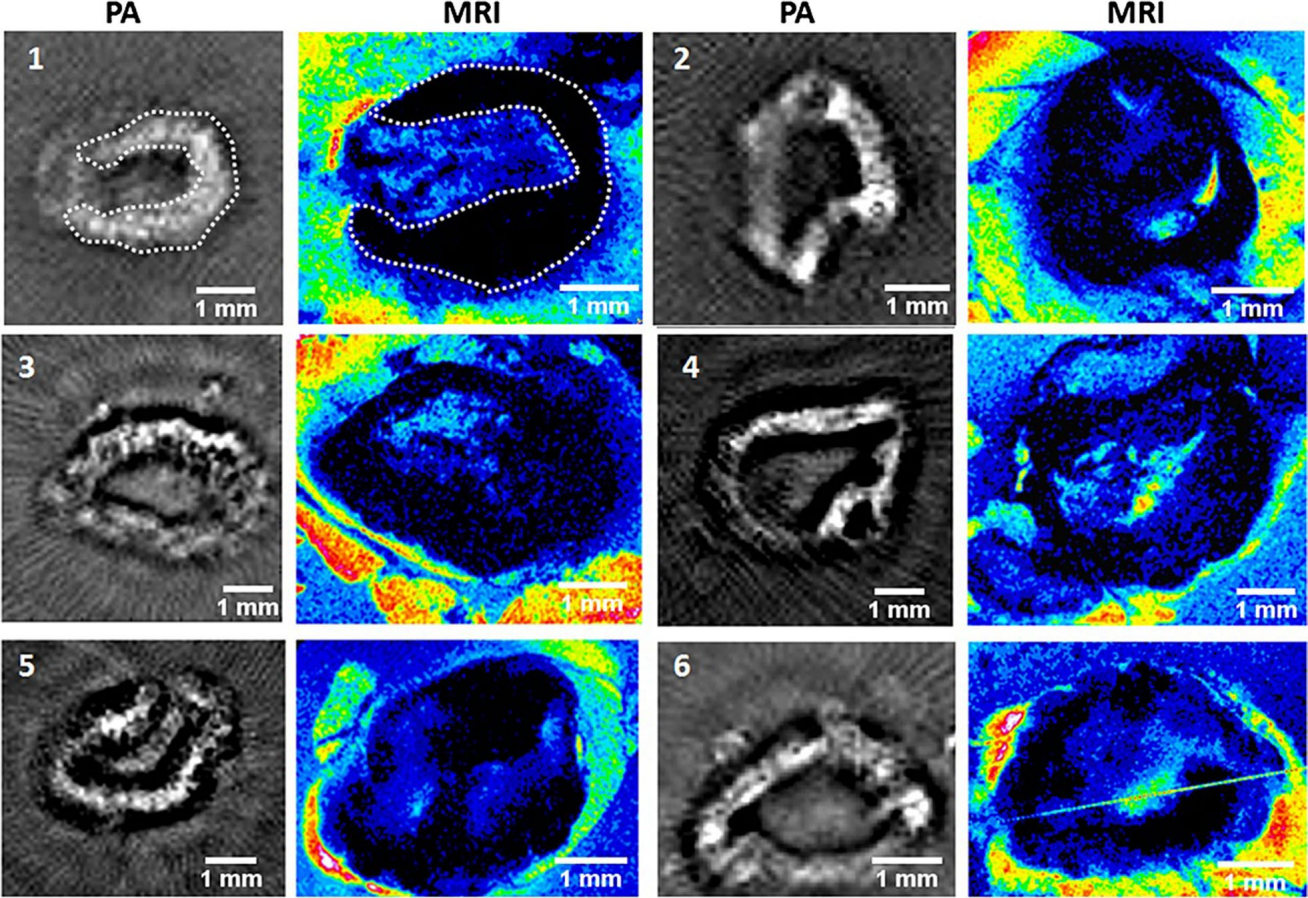
Photoacoustic and MR image comparison of all resected lymph nodes with contrast injection. As shown in lymph node 1 (white dotted line), the PA response pattern is comparable with the location of MRI signal decrease. Some nodes show a continuous contrast band throughout their periphery (1,5), while others show some small irregularities (4,6). The figure was reused from the research article published in the journal Contrast Media and Molecular Imaging by Grootendorst and co-workers in 2013 [190]

Nanoparticles based PA contrast agents has been very successful in vivo. Grootendorst and co-workers have successfully applied this technique in vivo and resected the lymphoma through lymph node imaging with iron oxide nanoparticles (IONPs) as the PA contrast agent (Fig. 10) [190]. IONPs coupled with NIR dye labelled peptides showed imaging and guided drug delivery for breast cancer management [191]. IONPs is a potential tool for multimodal imaging as the NIR tagging helps in PA based imaging and PTT, while the inherent paramagnetic property of IONPs aids in MRI based diagnosis. Together with the PAI and MRI based images a conclusive diagnosis of diseases with its severity, staging and prognosis can also be reported [106, 170, 192]. Live cell tracking at the lymphatic circulation with PA systems aids in intraoperative imaging, specifically to detect and remove the residual tumors after the resection [193]. Plasmonic nanobubbles tagged with biomolecules were reported to aid in highly sensitive detection of single residual tumors cells with real time 1 ms acoustic detection time and the signal reception from up to 4 mm depth [194]. Lapotko et al., had studied the ability of EGFR conjugated gold nanoparticles that can get accumulated specifically at the microtumor tissues and acoustic signals were detected from the gold NP targeted residual microtumors and cancer cells, which will then be resected easily [195].

### PAI guided drug delivery

Monitoring the delivery of drug through PAI is an upcoming field of research which showed significant success after intraoperative imaging, in the small animal level. The tissue specific delivery of drug is a key feature of an anti-cancer agent [196]. Engineering the molecules and formulation to achieve this are constantly being developed. However, controlled release of active drugs from these formulations are very challenging [197]. Several biomolecule-based targeting strategies were developed for site targeted and controlled delivery of drug [198]. On the other hand, success of optical and acoustic systems is significant in the development of cancer therapeutic strategies. The involvement of laser irradiation in PAI is a big advantage in drug delivery systems. Pulsed laser irradiation is an inherent trigger mechanism for drug release and at low laser intensity the site of delivery can be observed and located, while at higher laser intensity the controlled release of drug can be achieved [199].

**Fig. 11:**
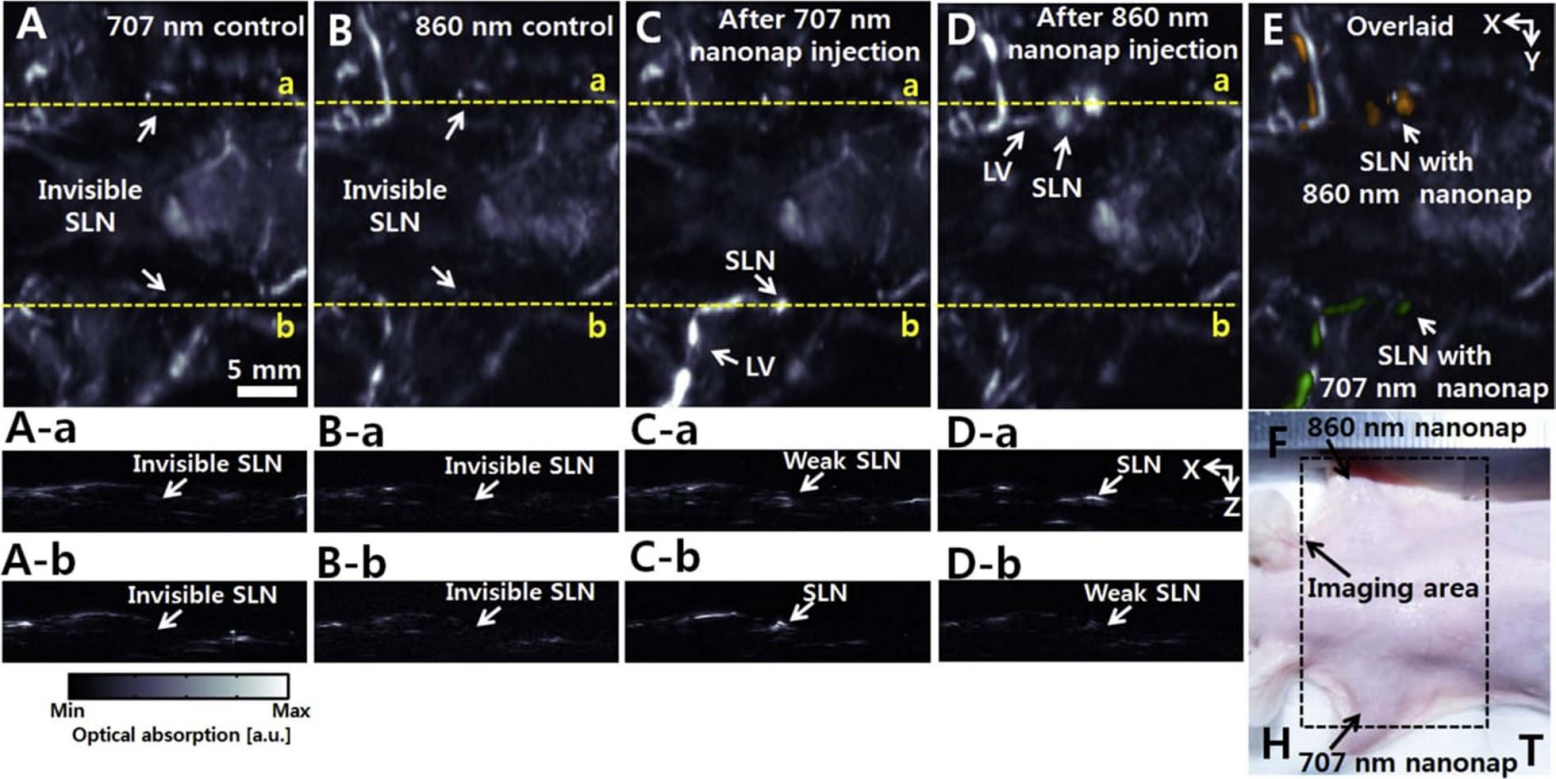
In vivo simultaneous dual-color PA imaging of a rat’s SLNs with injection of dual-color nanonaps (707 and 860 nm). **A** Control PA MAP image at 707 nm. **A-a**, **A-b** Control PA cross-sectional images of right and left axillary regions cut along the dotted lines a and b in **A**, respectively. **B** Control PA MAP image at 860 nm. **B-a**, **B-b** Control PA cross-sectional images of left and right axillary regions cut along the dotted lines a and b in **B**, respectively. 707 and 860 nm nanonaps (0.01 mL and 17.1 mg/mL) were injected simultaneously throughout the forepaw pads of both sides (i.e., left side; 707 nm nanonap and right side; 860 nm nanonap). **C** Post-injection PA image at 707 nm. **C-a**, **C-b** Corresponding PA cross sectional PA images cut along the dotted lines a and b in **C**. **D** Post-injection PA image at 860 nm. **D-a**, **D-b** Corresponding PA cross-sectional PA images cut along the dotted lines a and b in **D**. **E** Overlaid dual-color PA image of both right and left lymphatic systems. **F** Photograph of the dorsal region of the animal. *SLN* sentinel lymph node, *LV* lymphatic vessel, *H* head; and *T* tail. The figure was reused from the research article published in the journal Biomaterials by Lee, Lovell, Kim and co-workers in 2015 [202]

Gold nanocage, fucoidan capped gold nanoparticles, coupled with doxorubicin and paclitaxel loaded gold nanorods are the best examples of nanoformulations that aids in PA guided site targeted and controlled delivery of chemotherapeutic agents [116, 200, 201]. Currently the developments in lymphatic delivery of drugs and PAI based monitoring were not developed and are still in their budding stage. However, some studies showed promising results in lymphatic route of drug delivery guided by photoacoustic imaging [26]. Conjugation of biomolecules specific for lymphatic system with nanoparticles has aided the PAI lymphatic imaging and guiding the drugs for targeted delivery. Importance of lymphatic drug delivery is well-known based on the role of lymph nodes in cancer progression [193]. Nanoformulation of naphthalocyanines were successful in precision imaging of lymphatic system and individual patient-based cancer therapy (Fig. 11) [202]. Albumin conjugation to the chemotherapeutic drugs like paclitaxel and docetaxel were well studied for its enhanced cancer delivery [203]. When the hydrophobic domains of albumin were functionalized by NIR dyes such as indocyanine green, IR820, etc., the systemic stability was improved and becomes optimal for photoacoustic imaging of lymphatic system [142, 204]. Yucel and co-workers have developed a formulation consisting of non-fluorescent dye QC-1 conjugated albumin coupled with BODIPY-based fluorophore for PAI imaging of the lymphatic drainage in the regions from eyes to the neck region, which could be path breaking in theranostic imaging research as most of the metastasis originating from the head and neck cancers can be analyzed through the corresponding regional lymph nodes [142, 205].

## Conclusion and future prospects

**Fig. 12:**
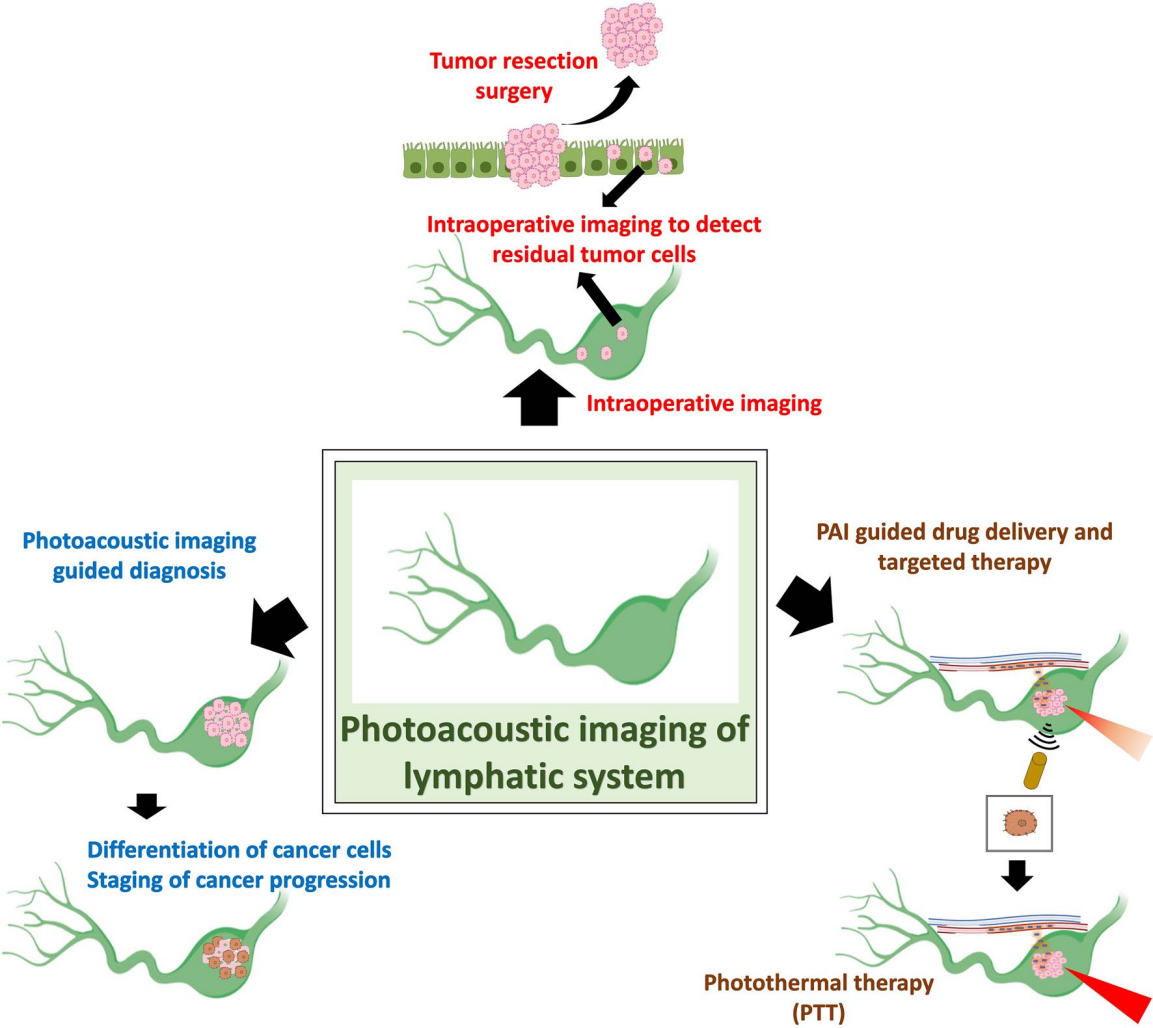
Summary of PAI of lymphatic system for cancer theranostics

Prevalence of cancer and the socio-economic burden it poses on the community is always on the rise. Since the discovery of the disease, new information on the basic biology behind the pathogenesis is being unearthed constantly which challenges the expert with its complexity, in addition to the unresolved concerns [206]. Nonetheless, the developments with respect to cancer management strategies are tremendous. Chemotherapeutic, physical, and biological treatment methods were employed and were reasonably successful only under specific conditions. The severity and stage of the disease progression play major part on deciding the optimal therapeutic strategy to be employed. Several upgradations of the current methods have resulted in successful management of cancer and even certain physical methods like radiation, US, photothermal effect and etc., combined with administration of chemotherapeutic agent had resulted in better efficiency [207]. However, the limitations like stability of drug, site targeted delivery and undesirable toxicity to the healthy tissue makes the management strategies challenging to the experts [129]. In the review we have discussed about the nanotheranostic agents in treatment of cancer guided by photoacoustic system. We detailed about the different nanoparticles that are used for PA applications and how they are targeted to the region of interest. The challenges faced by the scientists during development of photoacoustic imaging-based nanotherapy pointed at controlled release and site targeted activity as key areas to be explored for a better anti-cancer therapy. While differentiating normal and benign cells from metastatic cancer cells are the unaddressed concerns in the current diagnostic strategies. Hence, PAI of lymphatic system has shown significant improvement in that area specifically aiding in identifying the stage of cancer progression and metastasis orchestrated through the lymph nodes. Further the image guided surgery and drug delivery application of PAI was successful through the lymphatic route, but not yet translated for clinical applications as there are a lot of limitations like specificity, nano contrast agent accumulation and long-term toxicity, inability to cross lymph nodes and etc. [126]. Upgradation of lymphatic system imaging with PA systems devoid of such limitations and utilizing for cancer management might greatly help the clinicians in accurate diagnosis of cancer staging, where progression, metastasis and best possible treatment strategy can be reported (Fig. 12). The consistency with the diagnostic specificity and safer imaging methods makes PAI, the future of cancer theranostics. Many modifications to current methodology are possible and constant research on optimization might reduce the burden on clinicians and hospital care workers.

Future of PAI greatly depend on improving the challenges that are currently being unaddressed. Constant research and optimization will definitely make the PA based imaging modalities routinely used in the clinics. One of the major advancements required for PAI systems is identification of suitable and advanced computational system for image analysis. This will immensely help the clinicians as the currently available methods for image reconstruction algorithms and spectral un-mixing algorithms are very complex and time consuming [208]. Upgradation in PAI instrumentation has been very rapid and novel PA systems were developed recently such as PAE, hand held PAM and single-impulse panoramic photoacoustic computed tomography which will aid in acquiring images with high quality and better resolution by avoiding the interferences. Wearable PA devices are currently being studied for diagnosis of dental lesions [209–211]. PAI contrast agents with capacity to absorb longer wavelengths are preferred to overcome the challenges with deep tissue imaging [212]. Currently successful contrast agents that has arrived the clinical trials stages are Hb and melanin. The safety concerns, biological stability and evading reticulo-endothelial systems (RES) are some of the major concerns still threatens the chances of clinical translation for nanotheranostics used in imaging methods including PAI. Hence, current focus is on methods to engineer the nanomaterials to overcome such limitations [213]. Modifications of DNA nanocomplexes coupled with doxorubicin and a photosensitizer has successfully avoided the inclusion in the endosomes inside the cells [214]. The ability of PAI with intracellular/surface markers provides considerable potential in single cell imaging, which gives significant information in the cellular and molecular signaling pathways. Serious exploration in this regard will definitely help PAI methods to be utilized in clinics as many metabolic disorders can be successfully diagnosed with precision, other than cancer [215]. Lymphatic system has a significant edge over the other routes of nanomaterial administration and subsequent PAI imaging because of its high spatiotemporal resolution. Multimodal imaging systems are seriously considered for obtaining better imaging and conclusive evidences without overlapping of pathological symptoms [127, 129]. It was long before when the importance of lymphatic imaging was less sorted for disease management and with development of PAI, it is not far that a comprehensive imaging of whole body/regional lymphatic circulation will be a key method for management of complex disorders by aiding in timely diagnosis and precise treatment strategies.

## Data Availability

The data used to support this review are included within the article.
